# Targeted cell interconversions reveal inner hair cell control of organ of Corti cytoarchitecture

**DOI:** 10.1126/sciadv.adz3944

**Published:** 2025-10-29

**Authors:** Ignacio García-Gómez, Jemma L. Webber, Berta Soria-Izquierdo, John C. Clancy, Anne Duggan, Yingjie Zhou, Charles P. Murphey, Trevor D. M. Harriman, Jianghong Wang, Mary Ann Cheatham, Jaime García-Añoveros

**Affiliations:** ^1^ Department of Anesthesiology, Northwestern University Feinberg School of Medicine, Chicago, IL, USA.; ^2^ Hugh Knowles Center for Clinical and Basic Science in Hearing and its Disorders, Northwestern University, Evanston, IL, USA.; ^3^ Department of Communication Sciences and Disorders, Northwestern University, Evanston, IL, USA.; ^4^ Interdepartmental Neuroscience Graduate Program, Northwestern University, Chicago, IL, USA.; ^5^ Departments of Neuroscience and Neurology, Northwestern University Feinberg School of Medicine, Chicago, IL, USA.

## Abstract

The mammalian organ of Corti is a precisely intercalated mosaic of two types of mechanosensory hair cells (HCs) and six types of supporting cells (SCs) arranged in 11 parallel rows. Differentially specialized SCs surround inner HCs (IHCs) and outer HCs (OHCs). To elucidate the developmental roles of IHCs and OHCs in the formation, differentiation, and assembly of the various SCs, we genetically switched HC identities at several developmental stages and also generated mice lacking IHCs. We find that IHCs promote or induce the (i) differentiation of one SC type (outer pillar) at the expense of another (Deiters’), so that each completely and exclusively populates separate rows; (ii) packing density, but not identity, of inner pillar cells; and (iii) embryonic formation and (iv) cytoplasmic attraction of adjacent inner phalangeal cells, which envelop IHCs. Hence, developing IHCs dictate major aspects of SC identity and distribution to assemble the complex organ of Corti.

## Teaser

Genetically swapping identities between hair cell types reveal their role in assembling other cochlear cells during organogenesis.

## INTRODUCTION

The developmental formation of organs involves the coordinated placement of different types of cells with respect to one another. This complex assembly requires communication among the constituent cell types, with some instructing others how to differentiate and where to reside. Genetic and pharmacological studies have revealed many genes and molecular signaling pathways mediating these communications. While much can be inferred from these molecular studies about which cells send and receive particular signals, the complete effect that each cell type exerts on the others is rarely examined directly. The respective roles of different cell types could be elucidated by switching their fates or by preventing their production, preferably in an organ with precisely located cell types. Such conditions are met by the organ of Corti, the auditory sensory epithelium within the mammalian cochlea.

**Fig. 1.**
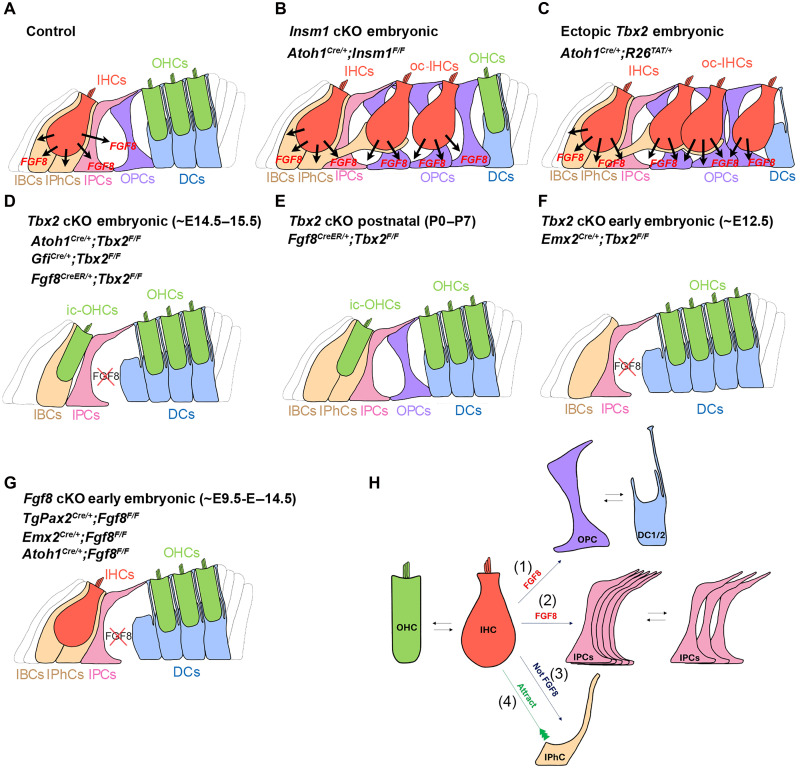
Summary of the developmental effects exerted by IHCs versus OHCs on the formation, distribution, and assembly of SCs of the organ of Corti. (**A** to **G**) Cartoons schematizing the cellular phenotypes of each of the mutants here examined, in which OHCs converted into outer compartment (oc)–IHCs, IHCs converted into inner compartment (ic)–OHCs, IHCs were absent, or IHCs were present but did not express FGF8. Effects on IPC density, depicted in (H), are not shown in these cross-sectional renditions because IPC identity is not affected. (**H**) Interconversions between OHCs and IHCs reveal four specific effects of IHCs on specific SC types. (1) Formation, via FGF8 signaling from IHCs, of OPCs at the expense of DC1/2s (but not DC3s), so that normally the ratio of SCs results in a single row of OPCs followed by three rows of DCs; (2) determination (also via FGF8 signaling emanating from IHCs) of abundance, but not identity, of IPCs, so that the IPC row accumulates IPCs at 1.5× the density of other SC and HC types; (3) formation of inner phalangeal cells (IPhCs), but not inner border cells (IBCs), during embryogenesis via signaling emanating from the IHCs that is not FGF8; (4) postnatal attraction of cytoplasmic extensions from IPhCs to envelop their basolateral sides. Pointed arrows represent inductive effects, and barbed arrows represent attractive effects.

The organ of Corti is a mosaic of highly specialized mechanosensory and supporting cells (SCs) distributed in a near-crystalline arrangement (Fig. 1A). In the medial or inner compartment (ic), a single row of inner hair cells (IHCs), endowed with prominent presynaptic structures, synapses onto most of the spiral ganglion neurons and serves as the prototypical sensory receptors, communicating sound information to the brain. In the lateral or outer compartment (oc), three (sometimes, four) rows of outer hair cells (OHCs), which are electromotile (*1*, *2*), serve as mechanical amplifiers, enhancing the detection of low-intensity sounds and the discrimination of sounds with similar frequencies. IHCs and OHCs are interspersed among highly specialized SCs adapted to their unique functions (*3*, *4*). IHCs are tightly enveloped by inner border cells (IBCs) and inner phalangeal cells (IPhCs), which take up the excess glutamate released by IHC presynaptic activity. OHCs are surrounded by Deiters’ cells (DCs), which provide support at their base and mechanically accommodate to their electromotility. Between the inner and outer rows of hair cells (HCs) stand the inner and outer pillar cells (IPCs and OPCs, respectively), which form a pivot that facilitates the optimal mechanical displacements of the organ in response to sound waves (*5*, *6*). The result is an intricate arrangement of highly specialized HCs and SCs required for the remarkable sensitivity and frequency selectivity of mammalian hearing.

We have a limited knowledge of how this elaborate supracellular structure is developmentally assembled (*7*). It is known that both HCs and SCs of the organ of Corti derive from precursor cells of the prosensory domain of the otocyst (*8*), a region that exits the cell cycle between embryonic day 12.5 (E12.5) at the cochlear apex and E14.5 at the base (*9*). The subsequent formation of alternating HCs versus SCs involves Notch-mediated lateral inhibition, whereby Notch ligands, Jagged2 and Delta-like-1, in some of the cells activate the Notch receptor in their immediate neighbors. Notch activation prevents cells from adopting an HC fate by repressing the expression of the master regulator atonal bHLH transcription factor 1 (*Atoh1*) (*10*–*12*). Thus, cells persistently expressing ATOH1 (those producing the ligands) will differentiate as HCs and their neighbors as SCs (*13*–*19*). During the postmitotic embryonic period from ~E14 to birth, mechanical forces and differential affinities presumably contribute through cell migrations and intercalations to align alternating HCs and SCs into parallel rows (*20*–*24*). How these rows end up consisting of exclusively one type of HC and/or SC remains unknown.

The distinct formation and differentiation of IHCs versus OHCs involves the gene regulatory factors, insulinoma-associated 1 (INSM1) and T-box transcription factor 2 (TBX2). INSM1, expressed in embryonic and early postnatal OHCs, consolidates their fate by preventing expression of a core set of early IHC genes, among which is *Tbx2* (*25*, *26*). In the absence of INSM1, nearly half of the nascent OHCs switch fates, express TBX2, and develop as IHCs (*26*–*28*). TBX2 is expressed by IHCs and many other cells of the cochlear epithelium but down-regulated in the cells that will give rise to OHCs and surrounding SCs (i.e., the outer compartment) (*28*, *29*). Despite its broad expression, TBX2 acts as a master regulator for the differentiation and maintenance of cochlear IHCs (*28*). Removal of TBX2 from embryonic IHCs results in their development as OHCs, and removal from postnatal IHCs results in their subsequent transdifferentiation into OHCs, replacing most IHC features and markers with those of OHCs. Conversely, ectopic expression of TBX2 in OHCs transdifferentiates them into IHCs (*28*–*30*). Hence, the presence or absence of TBX2 from a cochlear HC determines whether it becomes an IHC or OHC.

Regarding SCs, genetic and pharmacological experimentation reveals that fibroblast growth factor (FGF) receptor signaling is required for the formation of IPCs and OPCs and that the ligand and its source may be FGF8 produced by IHCs (*31*–*43*). However, direct tests of the IHC influence on pillar cells or other types of SC are yet to be performed. Here, we use genetic models in which, by manipulating *Insm1* and *Tbx2* expression, we switch the identity of IHCs and OHCs at various developmental stages or generate mice lacking IHCs. The first approach is conceptually analogous to classical transplantations, in which blocks of tissues are grafted elsewhere in the embryo and which revealed major multicellular organizers in development (*44*, *45*). However, the targeted cell interconversions that we developed afford an unprecedented precision for targeting individual cell types, rather than entire blocks of tissue. With these cell-identity changes, we examine the effects of two cell types (in this case, cochlear IHCs and OHCs) on the identity and distribution of other cells (in this case, six types of cochlear SCs) and, hence, on the assembly of a complex organ.

## RESULTS

**Fig. 2.**
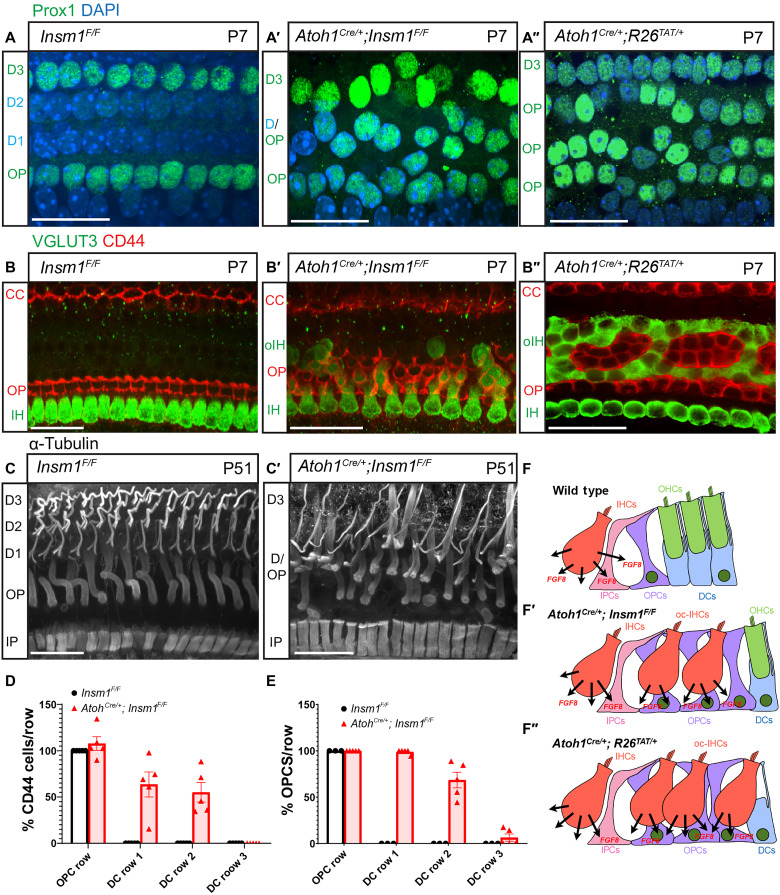
oc-IHCs induce formation of extra OPCs at the expense of DC1 and DC2, but not DC3. Immunolabeling on organs of Corti of [(A), (B), and (C)] controls (*Insm1^F/F^*), [(A′), (B′), and (C′)] HC-targeted cKOs of *Insm1* (*Atoh1^Cre/+^; Insm1^F/F^*; in which about half of the OHCs converted to oc-IHCs), and [(A″) and (B″)] HC-targeted expressors of ectopic TBX2 (*Atoh1^Cre/+^; R26^TAT/+^*, in which all OHCs converted to oc-IHCs). (**A**, **A′**, and **A″**) At P7, PROX1 expression is restricted to OPCs and DC3s of controls (A) but extends to cells in the position of DC1/2s in both mutants [(A′) and (A″)]. DAPI, 4′,6-diamidino-2-phenylindole. (**B**, **B′**, and **B″**) At P7, CD44 expression is restricted to OPCs and Claudius cells (CCs) in controls (B) but extends to cells in the position of DC1/2s in both mutants [(B′) and (B″)]. (**C** and **C′**) In adults, α-tubulin fills the thin DC phalanxes and thick OPC and IPC stems. While controls display one row of IPCs, one of OPCs, and three of DCs (C), the mutant has extra OPCs and correspondingly fewer DCs in DC rows 1 and 2. In these confocal projections, the apical portion of epithelium (reticular lamina) has been optically removed for phalanx versus stem visualization. (**D** and **E**) Quantifications in OPC and DC1 to DC3 rows of controls and HC-targeted cKOs of *Insm1* of CD44^+^ cells at P7 (D) and of adult OPCs and DCs as distinguished by their α-tubulin patterns (E). In mutants, about half of the cells in the position of DC1/2s, but not DC3, express OPC marker CD44 at P7 (D) and most of them look like OPCs as adults (E). (**F**, **F′**, and **F″**) Cell types in (F) controls, (F′) *Insm1* cKOs, and (F″) ectopic TBX2 expressors. Mutants have OHCs converted to oc-IHCs (half in *Insm1* cKOs and all in ectopic TBX2 expressors) and DC1/2s converted to OPCs. Green nuclei reflect PROX1 at P7. Scale bars, 20 μm.

We switched the identity of IHCs and OHCs at various times by manipulating either INSM1 or TBX2 as follows (Fig. 1, A to E): (i) By removing INSM1 conditionally with *Atoh1^Cre/+^; Insm1^F/F^*, about half of the OHCs express TBX2 and transdifferentiate into IHCs during embryogenesis, resulting in an outer compartment of cells with the features of IHCs (termed oc-IHCs) intermixed with OHCs (*26*, *27*). (ii) By ectopically expressing TBX2 in HCs starting ~E14.5 with *Atoh1^Cre/+^; R26^TAT^* (which permanently expresses TBX2 and an unstable form of ATOH1 in HCs upon Cre-mediated recombination of a “floxed” stop cassette), nearly all embryonic OHCs convert into oc-IHCs (Fig. 2B″) (*30*). (iii) By removing TBX2 from embryonic IHCs (at ~E14.5 in *Atoh1^Cre/+^; Tbx2^F/F^*, at ~E15.5 in *Gfi1^Cre/+^; Tbx2^F/F^*, or at the time of tamoxifen administration in *Fgf8^CreER/+^; Tbx2^F/F^*), nearly all HCs in the inner compartment develop as HCs with all examined features of OHCs [termed ic-OHCs; (*28*)]. (iv) By removing TBX2 from postnatal [postnatal day 0 (P0) to P9] IHCs (at the time of tamoxifen administration in *Fgf8^CreER/+^; Tbx2^F/F^*), nearly all HCs in the inner compartment develop as HCs with most features of OHCs [except the arrangement of apical stereocilia bundles, which remains IHC like; (*28*)]. (v) In addition, by removing TBX2 from the embryonic cochlear epithelium before the appearance of HCs (at ~E12 in *Emx2^Cre/+^; Tbx2^F/F^*), we generated mice with OHCs in the outer compartment but lacking IHCs or any HCs, in the inner compartment (Fig. 1F, described later here).

### OHCtooc-IHC conversion does not affect the outer versus inner compartment identity of SCs

Previous examination of *Insm1* conditional knockouts (cKOs), which have large numbers of oc-IHCs, revealed that SCs in the outer compartment did not display characteristics of those in the inner compartment (*27*). This was, in part, supported by the pattern of prospero homeobox 1 (PROX1) expression, a nuclear marker of IPCs, OPCs, and DCs of all three rows (DC1 to DC3) at P0. Here, we confirmed that neonatal *Insm1* cKOs displayed five rows of PROX1^+^ outer SCs (fig. S1, D and D′). Furthermore, IPCs, which may be immunolabeled in neonates with antibodies to neuropeptide Y (*46*) (NPY), nerve growth factor receptor P75/NGFRb (*32*, *39*, *40*), and angiotensin I–converting enzyme (ACE), formed a single row separating inner compartment and outer compartment in *Insm1* cKOs as in controls (fig. S1, A to C′). Last, IBCs and IPhCs, labeled with antibodies to fatty acid–binding protein 7 (FABP7) in neonates (fig. S1, E and E′) and to the glutamate transporter GLAST at later postnatal to adult stages (*27*), appeared under the IHCs, but not under the oc-IHCs of *Insm1* cKOs. As will be later described, we noticed postnatal GLAST^+^ extensions contacting the more medial oc-IHCs, but these were projections emanating from the IPhCs in the inner compartment, not due to conversion of oc-SCs into GLAST^+^ cells. All these results reveal that oc-IHCs do not induce surrounding oc-SCs to match their inner compartment type.

### Embryonic IHCs control the identity of OPCs versus DCs (DC1/2) via FGF8 signaling

Given ample evidence that FGF signaling likely emanating from IHCs induces pillar cell development (*31*–*42*, *43*), we first examined the effect of OHC–to–oc-IHC conversion on OPCs with several markers. At P7, PROX1 expression subsides in IPCs and DCs of rows 1 and 2 (DC1/2), so that it marks two separate rows of OPCs and DC3s, respectively (*47*). In HC-specific *Insm1* cKOs (*Atoh1^Cre/+^; Insm1^F/F^*), however, these intervening cells (between OPCs and DC3s) retained PROX1 expression, suggesting that they were not developing as DC1/2 (Fig. 2, A and A′). We also labeled postnatal OPCs with anti-CD44 (*38*). While, in controls, the CD44^+^ cells formed a single row, in *Insm1* cKOs, they formed up to three, labeling many cells that are normally DC1/2 (Fig. 2, B, B′, and D). Last, we distinguished adult PCs and DCs by their tubulin pattern, which forms thick, columnar stems in IPCs and OPCs (with differing inclinations and basal/nuclear positions at the inner and outer sides of the tunnel of Corti) and thin, wiry processes (called phalanxes) in the DCs (Fig. 2, C, C′, and E). *Insm1* cKOs displayed two to three extra rows of cells with OPC-like stems and only one to two rows of DC-like cells with phalanxes. Notably, cells in the DC3 row displayed phalanxes, not OPC-like stems (Fig. 2, C, C′, and E).

Similar results were obtained in cochleae with TBX2 ectopically expressed in embryonic HCs (*Atoh1^Cre/+^; R26^TAT/+^*), in which nearly all OHCs converted embryonically into oc-IHCs (Fig. 2B″) (*30*). Due to a clumping of oc-IHCs, which segregate from the SCs at their base, the cells are not arranged in rows. Nonetheless, by the same criteria (expression of PROX1 and CD44 at P7), it is apparent that all cells that should have been DC1 and DC2 display the markers of OPCs, while DC3s appear unaltered and aligned in a row at the lateral edge of the organ of Corti (Fig. 2, A″ and B″). We conclude that, in HC-specific *Insm1* cKO mice and in HC-specific TBX2 expressing mice, the excess IHC-like cells in the outer compartment (oc-IHCs) induced formation of OPCs at the expense of DC1 and DC2, but not D3 (Fig. 2, F to F″).

**Fig. 3.**
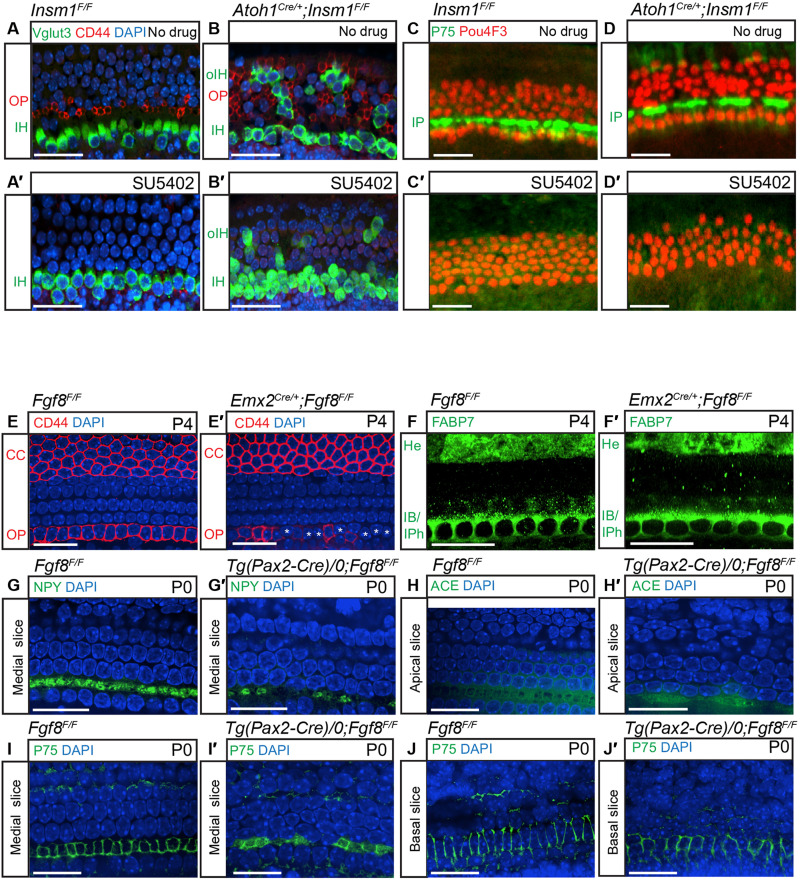
FGF8 signaling affects OPC identity and IPC density, but not identity. CD44 (OPCs), VGLUT3 (IHCs), P75 (IPCs), and POU4F3 (HCs) in organotypic cochlear explants (established at E17.5 and cultured for 6 days in vitro) of *Insm1^F/F^* [(control; (A), (A′), (C), and (C′)] and *Atoh1^Cre/+^; Insm1^F/F^* [(B), (B′), (D), and (D′)] exposed to FGFR inhibitor SU5402 [(A′), (B′), (C′), and (D′)]. (**A** to **B′**) FGFR inhibition prevents formation of normal and ectopic (oc-IHC induced) OPCs. *Insm1^F/F^* have one row of IHCs (IH) and one of OPCs (OP) (A), and *Atoh1^Cre/+^; Insm1^F/F^* have VGLUT3^+^ oc-IHCs and extra rows of CD44^+^ OPCs (B), while SU5402-exposed *Insm1^F/F^* (A′) and *Atoh1^Cre/+^; Insm1^F/F^* (B′) lack CD44^+^ OPCs (quantified in fig. S2). (**C** to **D′**) FGFR inhibition prevents formation of IPCs. *Insm1^F/F^* (control; C) and *Atoh1^Cre/+^; Insm1^F/F^* (D) have a row of P75^+^ IPCs (IP) between IHCs and OHCs, while SU5402-exposed *Insm1^F/F^* (C′) and *Atoh1^Cre/+^; Insm1^F/F^* (D′) lack P75^+^ IPCs. (**E** and **E′**) FGF8 promotes OPC formation. CD44^+^ CCs and OPCs on P4 cochleae of *Fgf8^F/F^* (control; E) and of *Emx2^Cre/+^; Fgf8^F/F^* (E′) with many CD44^−^ cells (asterisks) in the OPC row. (**F** and **F′**) FGF8 from IHCs or other cochlear epithelial cells is not required for IPhCs. Inner border and phalangeal (IB/IPh) and Hensen’s (He) cells’ marker FABP7 on P4 cochleae reveals FABP7^+^ IB/IPh cells around IHCs of both *Fgf8^F/F^* controls and *Emx2^Cre/+^; Fgf8^F/F^*. (**G** to **J′**) Cochlear FGF8 is required for the high density, but not identity, of IPCs. IPC markers (NPY, ACE, and P75) on neonatal cochleae of [(G), (H), and (I)] control (*Fgf8^F/F^*) and [(G′), (H′), and (I′)] cKOs that ablate *Fgf8* in all otocyst-derived cells, viewed at apical, medial, and basal/nuclear depths. Controls displayed continuous rows of IPCs at all depths [(G), (H), (I), and (J)]. cKO IPCs appeared discontinuous apically (H′) and medially [(G′) and (I′)], but continuous basally (J′). However, compared to controls IPC nuclei are loosely packed and wider in cKOs [(J) and (J′)]. Scale bars, 20 μm.

We then tested whether the DC1/2 to OPC conversion in *Insm1* cKOs was due to excess FGF signaling, given that oc-IHCs express FGF8 since E17.5 (*48*). We cultured organ of Corti explants from control and *Insm1* complete knockout (KO) embryos, dissected at E17.5 and kept in vitro for 6 days (to a developmental equivalent of ~P4). While, in these cultures, the rows of organ of Corti cells lose their straight alignment, control explants displayed an approximately single row of CD44^+^ OPCs close to the row of IHCs, while *Insm1* cKO explants displayed multiple CD44^+^ cells in the outer compartment under the oc-IHCs (Fig. 3, A and B, and fig. S2A). Addition of SU5402, an inhibitor of all four FGF receptors (FGFRs) (*49*), abolished the formation of CD44^+^ OPCs in both control and *Insm1* KOs (Fig. 3, A′ and B′, and fig. S2A). This result confirms that FGF signaling induces both the formation of the normal single row of OPCs (*31*, *33*, *39*), as well as the excess of OPCs (and concomitant reduction or absence of DC1 and DC2) observed in *Insm1* KOs.

Because IHCs express FGF8 throughout their embryonic and early postnatal development (up to P10 to P12), we sought to determine whether the OPC versus DC induction from IHCs is mediated by FGF8. We targeted *Fgf8* embryonically with *Emx2^Cre/+^; Fgf8^F/F^* [for ablation in the entire cochlear epithelium at ~E12; (*50*–*52*)]. This conditional deletion resulted in early postnatal lethality, but we were able to obtain one pup at P4 to examine the complement of CD44^+^ OPCs. These mutants displayed a great reduction of CD44^+^ OPCs (Fig. 3, E and E′). We conclude that the signal from IHCs that induces OPC versus DC differentiation is FGF8.

**Fig. 4.**
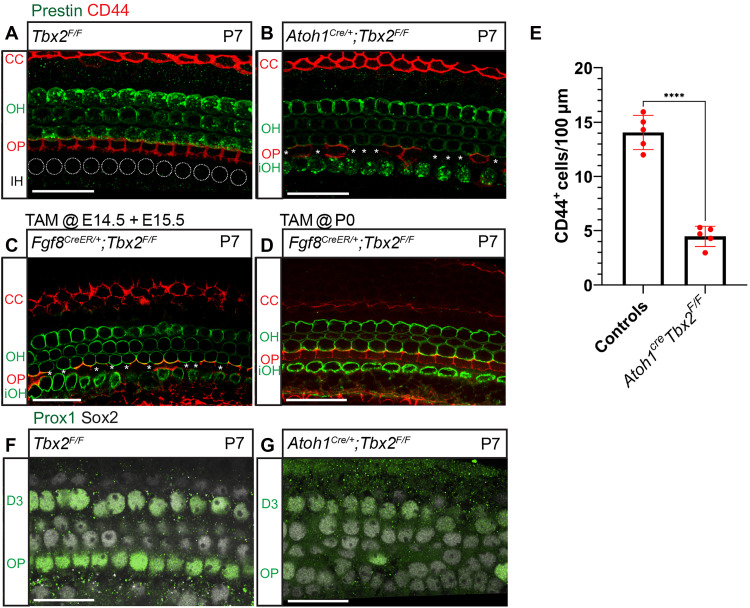
Embryonic, but not postnatal, conversion of IHCs to ic-OHCs reduces the number of OPCs postnatally. (**A** to **D**) CD44 (red), a marker of postnatal OPCs (OP) and CCs, and Prestin (green), a marker of OHCs (OH), examined in P7 organs of Corti. Controls [(*Tbx2^F/F^*; (A)] express CD44 in all cells in the OPC row (OP); dotted circles indicate the position of IHCs. In all *Tbx2* cKOs, IHCs have converted to ic-OHCs (iOH) and express Prestin. In addition, *Tbx2* cKOs targeted embryonically [(*Atoh1^Cre/+^; Tbx2^F/F^*; (B); *Fgf8^CreER/+^; Tbx2^F/F^* exposed to tamoxifen at E14.5 and E15.5; (C)] showed lack of CD44 cells (asterisks). In contrast, *Tbx2* cKOs targeted postnatally [(*Fgf8^CreER/+^; Tbx2^F/F^* exposed to tamoxifen at P0; (D)] express CD44 in all cells in the OPC row (OP), as in controls. TAM, tamoxifen. (**E**) Quantification of CD44^+^ cells in the OPCs row shows a reduction of OPCs in *Atoh1^Cre/+^; Tbx2^F/F^* (*n* = 5) versus controls (*n* = 5) (4.47 ± 0.92 versus 14.07 ± 1.57; ****P < 0.0001; unpaired *t* test). In cKOs, only 35.42 ± 8.25% of cells in the OPC row express CD44. (**F** to **G**) PROX1, at this time, a marker of OPCs (OP) and DCs of row 3 (D3), and of SOX2, a marker of all SCs, on the P7 organs of Corti of controls (*Tbx2^F/F^*; F) and of *Tbx2* cKOs targeted embryonically [(*Atoh1^Cre/+^; Tbx2^F/F^*; (G)], in which many cells in the OPC row (OP) are SOX2^+^, but PROX1^−^, corroborating the reduction of differentiating OPCs. Scale bars, 20 μm.

**Fig. 5.**
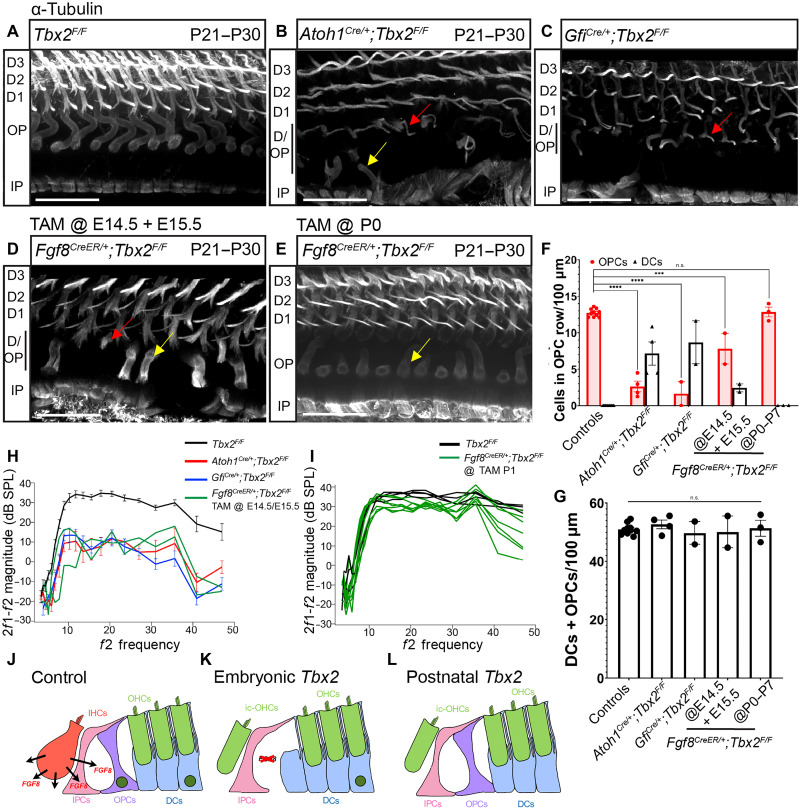
Embryonic, but not postnatal, IHCtoic-OHC conversion results in many OPC to DC conversions. (**A** to **E**) α-Tubulin in mature cochleae of control (A) and *Tbx2* cKOs targeted embryonically [(B) to (D)] and postnatally (E). Apical reticular lamina has been optically removed for process visualization. Controls have three DC rows (D1 to D3) of thin phalanxes and OPC (OP) and IPC (IP) rows of thick columnar stems. In contrast, *Tbx2* cKOs targeted embryonically contain many DC-like (red arrow) and OPC-like cells (yellow arrow) in the OPC row (D/OP). IPCs (IP) have thick columnar stems but are not aligned in a straight row. However, postnatal *Tbx2* cKOs (E) look like controls. Scale bars, 20 μm. (**F**) Quantification in the OPC row shows increase of DCs and reduction of OPCs in embryonic but not postnatal *Tbx2* cKOs. (**G**) Quantification of the total number of DCs plus OPCs shows no difference in the number of oc-SCs between control and the various cKOs. (**H**) DPOAEs of embryonic *Tbx2* cKOs (*n* = 12) are markedly reduced compared to controls (*n* = 13). Although *Atoh1^Cre/+^; Tbx2^F/F^* and *Gfi1^Cre/+^; Tbx2^F/F^* mice were not statistically different from one another, they differed from controls. Specifically, the *Atoh1^Cre/+^; Tbx2^F/F^* mice were statistically different from controls for *f*2 frequencies of greater than 6.8 kHz; the *Gfi1^Cre/+^; Tbx2^F/F^* mice were different from controls for *f*2 frequencies of greater than 3.4 kHz (*P* < 0.05 to *P* < 0.0001). (**I**) DPOAEs of postnatal *Tbx2* cKOs (*n* = 7) and littermate controls (*n* = 3) are similar. SPL, sound pressure level. (**J** to **L**) Cell types in controls (J) and embryonic *Tbx2* cKOs (K), in which IHCs converted to ic-OHCs embryonically and many OPCs have converted to DCs, and postnatal *Tbx2* cKOs [(induced at P0 to P7; (L)], in which IHCs converted to ic-OHCs postnatally but the OPCs are unaffected. Green nuclei (not shown for postnatal cKOs) reflect PROX1 at P7.

If FGF8-expressing IHCs induce the formation of OPCs at the expense of DCs, then we would expect that replacement of IHCs by OHCs would result in a lack of, or reduction in, OPCs and a concomitant increase in DCs. To test this prediction, we resorted to three embryonic cKOs of *Tbx2*, in which HCs in the position of IHCs develop embryonically with the features of OHCs, including a loss of *Fgf8* expression (*28*). In all three models (*Atoh1^Cre/+^; Tbx2^F/F^*, *Gfi1^Cre/+^; Tbx2^F/F^*, and *Fgf8^CreER/+^; Tbx2^F/F^* and tamoxifen at E14.5 and E15.5), there were many CD44-negative cells in the OPC row (Fig. 4, A to C and E), as well as many PROX1-negative nuclei (Fig. 4, F and G), at P7. Furthermore, in mature ears of these mutants, about half of the cells in the position of the OPCs lacked the columnar stems of the OPCs and displayed the thinner extensions (phalanxes) characteristic of DCs [Fig. 5, A to D and F; one-way analysis of variance (ANOVA) with Dunnett’s correction for multiple comparisons: control (12.74 ± 0.54; *n* = 10) versus *Atoh1^Cre/+^; Tbx2^F/F^* (2.63 ± 1.44; *n* = 3; ****P < 0.0001), versus *Gfi1^Cre/+^; Tbx2^F/F^*(1.63 ± 2.32; *n* = 2; ****P < 0.0001), and versus *Fgf8^CreER/+^; Tbx2^F/F^* and tamoxifen at E14.5 and E15.5 (7.85 ± 2.98; *n* = 2; ***P < 0.001)]. Because the DC phalanxes and the OPC stems projected in opposite directions and did not align in a clear row, we wondered whether there might be a loss of OPCs rather than a conversion into DCs. However, we counted all cells (DCs and OPCs) in the *Tbx2* cKOs and found that the sum of both cell types did not differ from that of controls {Fig. 5G; one-way ANOVA with Tukey correction for multiple comparisons resulting in no statistically significant differences between any group [not significant (n.s.)]: control (51.06 ± 1.85; *n* = 10) versus *Atoh1^Cre/+^; Tbx2^F/F^* (52.72 ± 2.95; *n* = 3), versus *Gfi1^Cre/+^; Tbx2^F/F^*(49.79 ± 5.56; *n* = 2), versus *Fgf8^CreER/+^; Tbx2^F/F^* and tamoxifen at E14.5 and E15.5 (50.13 ± 7.69; *n* = 2), and versus *Fgf8^CreER/+^; Tbx2^F/F^* and tamoxifen at P0 to P7 (51.41 ± 4.70; *n* = 3)}, disproving a net loss of OPCs. Hence, replacing embryonic IHCs by ic-OHCs resulted in the development of DCs in place of many of the OPCs (Fig. 5, J and K).

To determine whether OPC induction by IHCs is restricted to a period of development, we ablated *Tbx2* postnatally by administering tamoxifen to *Fgf8^CreER/+^; Tbx2^F/F^* pups after birth (Figs. 3E and 4E). As previously shown, this induces the transdifferentiation of nearly all IHCs into OHC-like cells (ic-OHCs). In these postnatal cKOs, OPCs and DCs formed normally and in the expected ratios, as demonstrated by the single continuous row of CD44^+^ cells at P7 (Fig. 4D) and by the single row of OPCs plus three rows of DCs revealed in adult ears with tubulin labeling [Fig. 5, E, F, and L; control (12.74 ± 0.54; *n* = 10) versus *Fgf8^CreER/+^; Tbx2^F/F^* and tamoxifen at P0 to P7 (12.85 ± 1.18; *n* = 10; n.s., *P* > 0.05; one-way ANOVA with Dunnett’s correction for multiple comparisons)]. As further evidence of the structural differences between the embryonically and postnatally generated *Tbx2* cKOs, we compared their distortion product otoacoustic emissions (DPOAEs), which do not require IHCs but rely on a structurally normal outer compartment. While embryonically generated *Tbx2* cKOs displayed severely reduced DPOAEs (Fig. 5H), as may be expected by their replacement of many OPCs by DCs and their malformed tunnel of Corti, postnatally generated cKOs had near normal DPOAEs (Fig. 5I), as expected from their intact tunnel of Corti and outer compartment. Together, we conclude that the induction of OPC (versus DC) by IHCs occurs during an embryonic and perhaps early postnatal period, but not later.

### Embryonic IHCs determine the density, but not the identity, of IPCs via FGF8 signaling

We next examined the IPCs, which, in neonates, are labeled with antibodies to P75, NPY, and ACE and which, in adults, are characterized by their columnar tubulin stems. In addition, the row of IPCs contains a greater number of cells (~1.5×) than those of OPCs and DCs (*53*), so that their nuclei are compacted and adopt a characteristic oblong (as viewed from the top) shape. In *Insm1* cKOs, the row of IPCs was unaltered by all these criteria, and there were no IPC-like cells under the ectopic oc-IHCs (fig. S1, A to D′). This implies that (i) oc-IHCs cannot induce precursors of oc-SCs (OPCs and DCs) to differentiate into IPCs and (ii) the row of IPCs does not appear to be affected by the additional oc-IHCs.

**Fig. 6.**
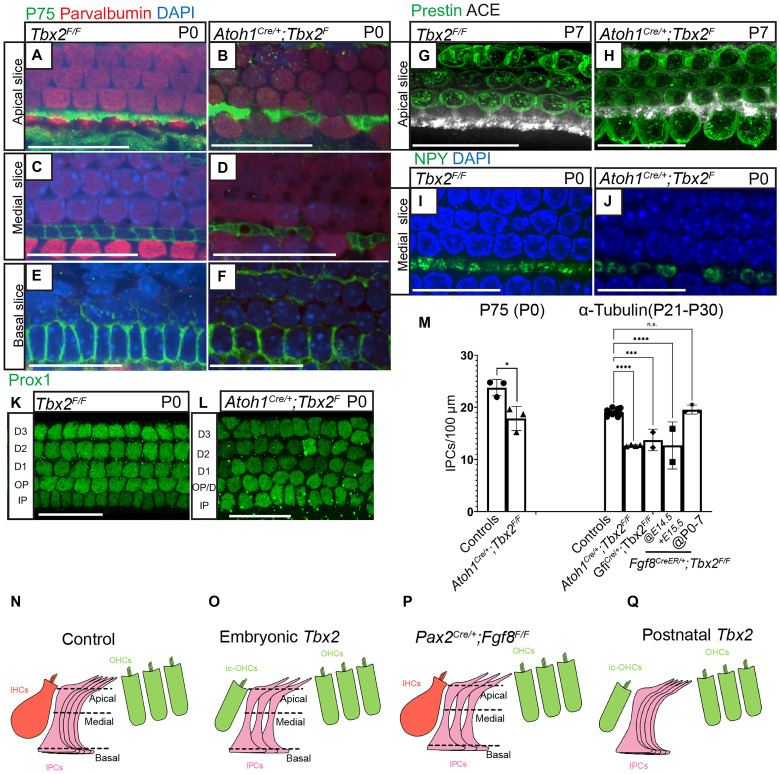
Embryonic conversion of IHCs to ic-OHCs reduces density but does not affect identity of IPCs. (**A** to **F**) Neonatal IPC marker P75 and HC marker Parvalbumin in cochleae of controls [(A), (C), and (E)] and *Tbx2* cKOs [(B), (D), and (F)], imaged at apical, middle, and basal/nuclear depths. Controls display continuous P75^+^ IPCs at all depths [(A), (C), and (E)] and densely packed, oblong-shaped nuclei basally (E). *Tbx2* cKOs display discontinuous P75^+^ IPCs [(B) and (D)] at apex and middle but basally a continuous line of P75^+^ IPCs with less packed, cuboidal nuclei (F). (**G** and **H**) ACE, an apical IPC marker and Prestin, an OHC marker, in P7 cochleae of controls and *Tbx2* cKOs. Controls display continuous ACE^+^ IPCs medial to OHCs. *Tbx2* cKOs have gaps of ACE^+^ IPCs between OHCs and ic-OHCs. (**I** and **J**) NPY, an apical and medial IPC marker, with nuclear DAPI, in cochleae of P0 controls and *Tbx2* cKOs. Controls show continuous NPY^+^ IPCs. *Tbx2* cKOs show gaps in the NPY^+^ IPC row. (**K** and **L**) PROX1 at P0 of controls and *Tbx2* cKOs. Both express PROX1 in five SC rows, but IPCs are less packed and cuboidal in *Tbx2* cKOs. (**M**) IPC quantification based on P75 expression at P0 or α-tubulin pattern at P21 to P30 (as in Fig. 5). Both revealed an IPC reduction in embryonic, but not postnatal, *Tbx2* cKOs. (**N** to **Q**) Cell types in control (N), embryonic *Tbx2* cKO (O), embryonic *Fgf8* cKO (P; summarizing Fig. 3, G to J′), and postnatal *Tbx2* cKO (Q). In both embryonic *Tbx2* cKOs, in which IHCs converted to ic-OHCs embryonically, and *Fgf8* cKOs, in which IHCs do not transform to oc-IHCs but fail to express FGF8, IPCs appear fully differentiated but at low density. In postnatal *Tbx2* cKOs, in which IHCs converted to ic-OHCs after birth, IPCs appear fully differentiated and at their characteristically high density. Scale bars, 20 μm.

We next considered whether the resident IHCs (those in the inner compartment) influence the formation of IPCs. We addressed this by converting IHCs into ic-OHCs at different stages of development. In embryonically generated *Tbx2* cKOs, single rows of P75^+^, NPY^+^, and ACE^+^ IPCs were detected in neonates (Fig. 6, A to J), although some gaps with no immunoreactivity were apparent. In adults labeled with α-tubulin, a row of IPC-like cells was also visible, again with gaps between the cells (Fig. 5, A to D, I, and J). Examination of neonatal IPCs at different depths in the epithelium revealed these gaps at their apical ends and middle stems (Fig. 6, A to D). However, at the level of their nuclei (basal portion of the cell), the IPCs aligned into a continuous row, but they did not have the oblong shape and concomitant higher density of controls (Fig. 6, E and F). This change in shape and density was also revealed by the nuclear marker PROX1 (Fig. 6, K and L). The expanded, more cuboidal nuclei of *Tbx2* cKOs can be explained by their reduced density. While a characteristic of the IPC row is a higher (~1.5) density than that of the OPC and DC rows, in *Tbx2* cKOs, the density of IPCs was reduced to about the same as that of OPCs and DCs. The gaps observed at the middle and apical levels in the IPC row of *Tbx2* cKOs are also consistent with the lower density of IPCs, because their columnar stems are not wide enough to form a continuous palisade as in the densely packed wild type controls. The density of IPCs, whether quantified postnatally with P75 labeling [Fig. 6M; control (23.8 ± 1.59; *n* = 3) versus Atoh*1^Cre/+^; Tbx2^F/F^* (17.85 ± 2.3; *n* = 3; *P < 0.05; unpaired *t* test)] or in adults with α-Tubulin labeling [Fig. 6M; control (19.15 ± 0.68; *n* = 12) versus *Atoh1^Cre/+^; Tbx2^F/F^* (12.68 ± 0.15; *n* = 4; ****P < 0.0001), versus *Gfi1^Cre/+^; Tbx2^F/F^*(13.75 ± 2.08; *n* = 2; ***P < 0.001), and versus *Fgf8^CreER/+^; Tbx2^F/F^* and tamoxifen at E14.5 and E15.5 (12.69 ± 4.5; *n* = 2; ****P < 0.0001; one-way ANOVA with Dunnett’s correction for multiple comparisons)], was reduced in embryonically generated *Tbx2* cKOs (Fig. 6, N and O). In postnatally generated *Tbx2* cKOs, the row of IPCs formed a continuous palisade at high density like those of controls [Figs. 5E and 6, M and Q; control (19.15 ± 0.68; *n* = 12) versus *Fgf8^CreER/+^; Tbx2^F/F^* and tamoxifen at P0 to P7 (19.55 ± 0.85; *n* = 3; n.s., *P* > 0.05; one-way ANOVA with Dunnett’s correction for multiple comparisons)]. These results suggest that IHCs, while not required for the identity of IPCs (which express all four markers examined in mutants with ic-OHCs in place of IHCs), are required during embryogenesis for the higher number and dense packing of IPCs.

Because blocking FGFRs (and other growth factor receptors) with SU5402 in cochlear explants prevented the formation of IPCs [as assessed from the lack of P75^+^ cells in Fig. 3 (C to D′) and previously reported in (*32*)] and because IPCs in *Fgfr3* KOs failed to differentiate (*31*, *34*, *35*), we next addressed whether the increase of IPC numbers could be due to FGF8 signaling from the IHCs. We targeted *Fgf8* embryonically with *Tg(Pax2-Cre)/0; Fgf8^F/F^* [for ablation in the entire otocyst by E9.5; (*54*)]. In these *Fgf8* cKOs, rows of P75^+^, NPY^+^, and ACE^+^ IPCs formed, but with apparent gaps in their apical and middle positions (Fig. 3, G to I′) and with a continuous row of cuboidal nuclei without the oblong shape and high-density characteristic of controls (Fig. 3, J and J′), as in the embryonic cKOs of *Tbx2* (Fig. 6F). This lower density of IPCs is not due to a difference in cochlear length, which does not differ between cKOs and controls (fig. S2), something that could have resulted from a defect in convergence extension. Hence, FGF8 signaling from IHCs is required for the high density of IPCs and for the consequences that this packing has on their shape (Fig. 6P). However, based on the expression of three IPC markers (P75, ACE, and NPY), neither FGF8 from otocyst-derived cells nor IHCs seem to be required for the developmental identity of IPCs. The differentiation of these cells must be induced by signaling mechanisms or morphogens emanating from cells other than IHCs.

### IHCs attract cytoplasmic extensions from, and are required for the formation of IPhCs, but not IBCs

IBCs and IPhCs are the ic-SCs that wrap around the IHCs. Both express the same known markers and cluster together in single-cell transcriptomic studies (*55*), so they are, to date, molecularly indistinguishable. In mature cochleae, their nuclei are not aligned in straight rows, unlike the other cells in the organ of Corti. Hence, they can only be distinguished by their cytoplasmic extensions surrounding the IHCs: IBCs contact their medial side, separating IHCs from cells of the greater epithelial ridge or Kölliker’s organ of neonates; IPhCs contact their lateral side, separating IHCs from IPCs. IBCs and IPhCs can be labeled with antibodies to the FABP7 in neonates (*56*) and to the glutamate transporter (GLAST) in later postnatal and mature ears (*56*–*58*).

**Fig. 7.**
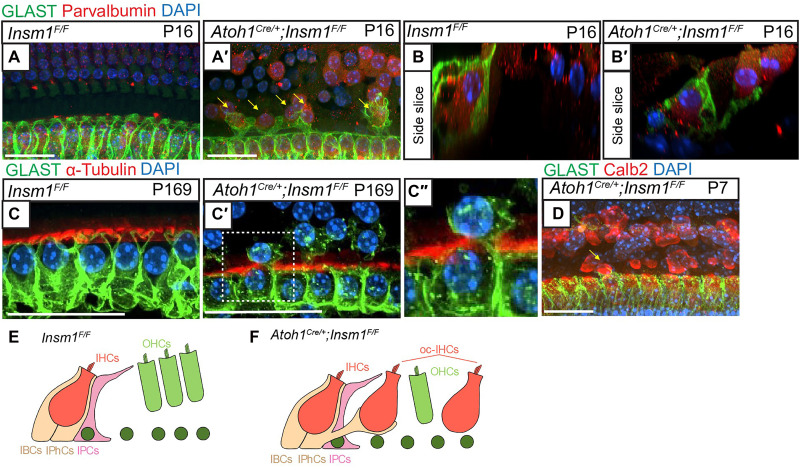
Postnatal oc-IHCs attract cytoplasmic extensions from inner compartment IPhCs. (**A** to **B′**) Immunolabeling of GLAST (green), a marker for IBCs and IPhCs, and Parvalbumin (red), a marker of IHCs, in the P16 organs of Corti of (A) controls (*Insm1^F/F^*) and (A′) HC-targeted cKOs of *Insm1* (*Atoh1^Cre/+^; Insm1^F/F^*; in which about half of the OHCs have converted to oc-IHCs). While, in controls, GLAST^+^ cells (IBCs and IPhCs) are seen only around IHCs, in cKOs, the IPhCs extend projections into the outer compartment and contact oc-IHCs (yellow arrows). (B) and (B′) Side view of (A) and (A′) showing (B) IHCs wrapped by IBCs and IPhCs in the control and (B′) cytoplasmic projection of GLAST^+^ IPhCs into the outer compartment and contacting oc-IHCs. (**C** to **C″**) Immunolabeling of GLAST (green), a marker of IBCs and IPhCs, and α-tubulin (red), which labels the palisade of IPCs, in the adult (P169) organs of Corti of (C) controls (*Insm1^F/F^*) and (C′) *Atoh1^Cre/+^; Insm1^F/F^* [magnified in (C″)]. A GLAST^+^ cytoplasmic projection from an IPhC crosses through the IPC row and contacts an oc-IHC. (**D**) Immunolabeling of GLAST (green) and Calb2 (a marker of IHCs and oc-IHCs; red), in the P7 organs of Corti of *Atoh1^Cre/+^; Insm1^F/F^* showing only one cytoplasmic projection (yellow arrow) toward one of several oc-IHCs. (**E** and **F**) Schematic summary of the cell types in the organ of Corti of (E) controls (*Insm1^F/F^*), in which IBCs and IPhCs wrap around IHCs and do not project to the outer compartment. The green nuclei represent the PROX1^+^ (at P0) DCs (DC1, DC2, and DC3), OPCs, and IPCs; (F) *Insm1* cKOs (*Atoh1^Cre/+^; Insm1^F/F^*), in which the oc-SCs do not become IPhCs (still PROX1^+^) but the IPhCs from the inner compartment extend projections through the row of IPCs into the outer compartment and contact oc-IHCs.

In neonatal *Insm1* cKOs, we detected FABP7^+^ SCs around IHCs, but not around oc-IHCs (fig. S1, E and E′). As before (*27*), in older *Insm1* cKOs, we detected GLAST^+^ SCs around IHCs, but not in the SCs of the outer compartment, those with nuclei under the OHCs and oc-IHCs. However, we noticed as early as P7 GLAST^+^ cytoplasmic extensions protruding from the base of the IPhCs in the inner compartment, crossing into the outer compartment by squeezing between the IPCs and contacting the closest oc-IHCs (Fig. 7, A to F). Most of these extensions form postnatally, increasing in frequency from P7, when ~12% of the IPhCs extend small projections (Fig. 7D) to P16, when 40% IPhCs extend even larger projections (Fig. 7A′). Hence, while oc-IHCs do not induce formation of IBCs/IPhCs from SC precursors of the outer compartment, they attract cytoplasmic extensions from the IPhCs in the inner compartment to contact them (Fig. 7, E and F).

**Fig. 8.**
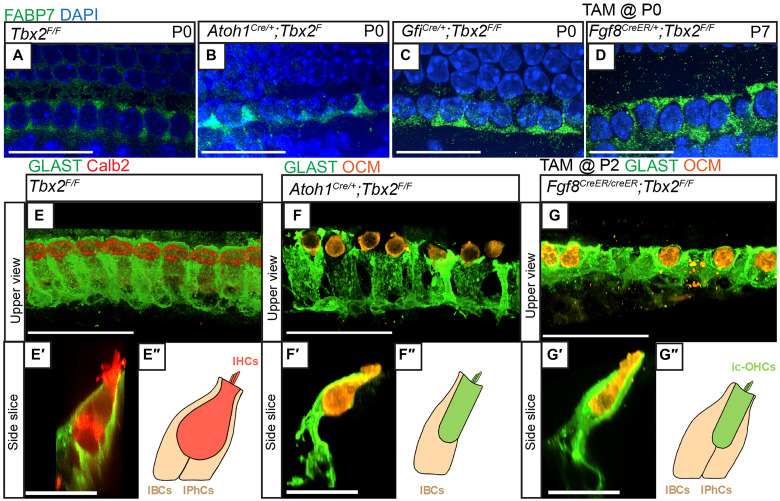
Embryonic IHC to ic-OHC conversion results in lack of IPhCs. (**A** to **D**) IBC and IPhC marker FABP7 in the postnatal (P0 or P7) cochleae of (A) controls (*Tbx2^F/F^*) and *Tbx2* cKOs targeted embryonically with (B) *Atoh1^Cre/+^; Tbx2^F/F^* and (C) *Gfi1^Cre/+^*;*Tbx2^F/F^* or postnatally with (D) *Fgf8^CreER/+^; Tbx2^F/F^* injected with tamoxifen at P0. Control shows FABP7^+^ cytoplasmic staining around medial and lateral sides of IHCs (A). Embryonic *Tbx2* cKOs show FABP7^+^ cytoplasmic staining on the medial, but not lateral, sides of ic-OHCs [(B) and (C)]. However, postnatal *Tbx2* cKOs (targeted at P0 and euthanized at P7) show FABP7^+^ cytoplasmic staining around medial and lateral sides of ic-OHCs (D). (**E** to **G″**) Mature IBC and IPhC marker GLAST, IHC marker Calb2, and ic-OHC marker Oncomodulin (OCM) in adult cochleae of [(E) and (E′)] controls and of *Tbx2* cKOs targeted embryonically with [(F) and (F′)] *Atoh1^Cre/+^; Tbx2^F/F^* and postnatally with [(G) and (G′)] *Fgf8^CreER/+^; Tbx2^F/F^* injected with tamoxifen at P2. [(E) to (E″)] Longitudinal (E) and radial (E′) confocal projections of controls show GLAST^+^ cytoplasmic extensions wrapping around Calb2^+^ IHCs. See movie S1 for a three-dimensional (3D) visualization and fig. S4 for quantification. (E″) Interpretation: IBCs and IPhCs wrap around the medial to lateral sides of IHCs. [(F) to (F″)] Longitudinal (F) and radial (F′) confocal projections of embryonic *Tbx2* cKOs show GLAST^+^ cytoplasmic extensions wrapping around medial, but not lateral, sides of OCM^+^ ic-OHCs. See movie S2 for a 3D visualization. (F″) Interpretation: IBCs wrap around the medial side of ic-OHCs but IPhCs are missing. [(G) to (G″)] Longitudinal (G) and radial (G′) confocal projections of postnatal *Tbx2* cKOs show GLAST^+^ cytoplasmic extensions wrapping around OCM^+^ ic-OHCs. (G″) Interpretation: IBCs and IPhCs wrap around the medial and lateral sides of ic-OHCs, as in controls. Scale bars, 20 μm.

We then proceeded to examine what effects the conversion of IHCs to ic-OHCs may have on IBCs and IPhCs. In embryonic cKOs of *Tbx2*, the FABP7^+^ (visible in neonates) and GLAST^+^ (visible at later postnatal and mature stages) IBCs and IPhCs did not wrap completely around the ic-OHCs, as they do around IHCs in controls. Instead, these cells contacted the medial, but not lateral, sides of ic-OHCs (Fig. 8, A to C and E to F′, and movies S1 and S2). Quantification of the nuclei of GLAST^+^ cells revealed that, in controls, there are 30.00 ± 4.93 (SD) cells/100 μm (*n* = 6). By contrast, embryonic *Tbx2* cKOs contained only 12.72 ± 0.48 (SD) cells/100 μm (*n* = 4), roughly half as many. While, based on nuclear position, we cannot distinguish which cells are present or absent, based on their extensions on the medial side of the IHCs, we identify the cells remaining as IBCs and the missing ones as IPhCs (Fig. 8, E″ and F″).

By contrast, postnatal cKOs of *Tbx2* (induced at P0 to P2) contained FABP7^+^ and GLAST^+^ IBCs and IPhCs wrapping fully around many of the ic-OHCs, as they do around the resident IHCs of control mice (Fig. 8, D and G to G″). We conclude that IHCs are required for the formation or maintenance of some of the ic-SCs (the IPhCs), but not others (the IBCs), and that this requirement might be largely restricted to an embryonic period of development.

We next wondered whether the effect of IHCs on IPhCs was mediated by FGF8, as is the effect on OPCs and IPCs. Because the *Fgf8* cKOs died shortly after birth, we could examine their IBCs and IPhCs only in neonates with FABP7. However, it became evident that, in these cKOs lacking FGF8 from IHCs (and from any other cell in the cochlear epithelium of *Emx2^Cre/+^; Fgf8^F/F^*), a normal complement of IBCs and IPhCs completely surrounded the basolateral surfaces of all IHCs (Fig. 3, F and F′). Hence, while IHCs are needed for the generation or persistence of IPhCs, this effect does not require FGF8 and must involve another signaling mechanism.

### IPCs, IBCs, and some OPCs are generated in the complete absence of IHCs

**Fig. 9.**
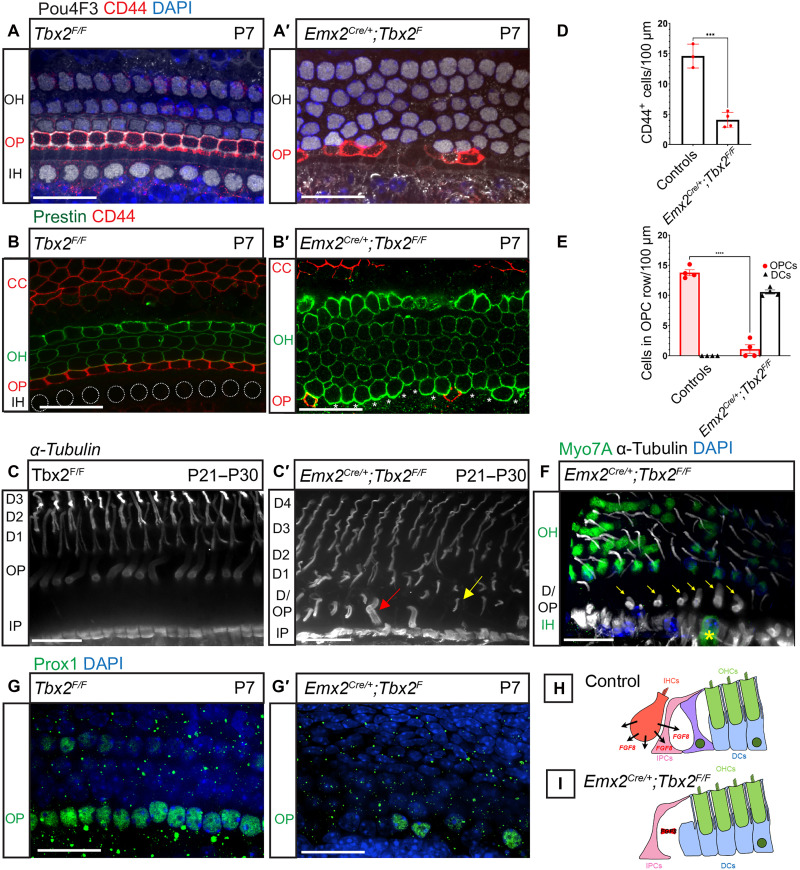
Lack of IHCs results in conversion of most, but not all, OPCs to DCs. (**A** to **B′**) Postnatal OPC (OP) and CC marker CD44, HC marker POU4F3, and OHC marker Prestin in P7 cochleae of *Tbx2^F/F^* (controls) and *Emx2^Cre/+^; Tbx2^F/F^*. Controls show CC and a continuous row of CD44^+^ OPCs (OP) separating three rows of OHCs (OH) from one row of IHCs (IH; marked with dotted circles in B). *Emx2^Cre/+^; Tbx2^F/F^* show a discontinuous row of CD44^+^ OPCs separating an outer compartment with four to five rows of OHCs from an inner compartment with no HCs (IHCs or ic-OHCs). Asterisks indicate CD44^−^ cells in the OPC row (B′). (**C** and **C′**) α-Tubulin, which reveals differing thicknesses of DCs and PCs, in mature cochlea of controls and cKOs. Apical reticular lamina is optically removed for visualization. In controls, DCs (D1 to D3) show their characteristic thin phalanges in three rows, whereas OPCs (OP) and IPCs (IP) display thicker, columnar stems. cKOs display four DC rows (D1 to D4) and, in the OPC row (D/OP), many cells with DC-phalanx-like processes (yellow arrow) and few OPC stem–like processes (red arrow). (**D**) Quantification of CD44^+^ OPCs shows their reduction in cKOs. (**E**) Quantification reveals DC increase and concomitant OPC reduction in mature cKOs. (**F**) Mature cKO cochlea showing one remaining IHC (Myo7A^+^) medial to a stretch of six OPCs (yellow arrows). (**G** and **G′**) PROX1, at this time, and position a marker of OPCs (OP), in the P7 basal cochleae of (G) controls, with continuous PROX1^+^ OPCs, and (G′) cKOs, with many PROX1^−^ cells in the OPC row. (**H** and **I**) Cell types in controls and in *Emx2^Cre/+^; Tbx2^F/F^*, which lack IHCs, have extra rows of OHCs and underlying DCs and have most OPCs converted to DCs. Green nuclei reflect PROX1 at P7. Scale bars, 20 μm.

Because the embryonic removal of *Tbx2* used above is triggered by Cre expression under the control of HC (*Atoh1* or *Gfi1*)– or IHC (*Fgf8*)–specific promoters, we suspected that, in these mice, nascent IHCs would be generated before their conversion to ic-OHCs. This was confirmed in the earliest of these cKOs (*Atoh1^Cre/+^; Tbx2^F/F^*) by performing in situ hybridization with the earliest known marker of IHCs, *Fgf8* mRNA. At E17.5, when the basal HCs have been differentiating for ~2 days and apical HCs are initiating their differentiation, both basal and apical IHCs of control cochleae express *Fgf8*. By contrast, in *Tbx2* cKOs at this same stage, basal HCs in the position of IHCs no longer express *Fgf8* (because they have converted, or are converting, into ic-OHCs), but apical and, hence, less differentiated IHCs still express *Fgf8*, albeit at weaker levels (fig. S3). We wondered whether these early IHCs transiently present in embryonic *Tbx2* cKOs could have induced the formation of the few OPCs (35.4%; Fig. 5F) that do not convert into DCs, as well as of the many apparently normal IPCs and IBCs. Therefore, we aimed at ablating *Tbx2* before the developmental onset of HCs. For this, we generated a novel mouse model, *Emx2^Cre/+^; Tbx2^F/F^*. In these embryos, we never detected *Fgf8*-expressing cells in the position of HCs (fig. S3), consistent with a lack of IHCs from the onset of cochlear development. Unexpectedly, examination of postnatal and adult *Emx2^Cre/+^; Tbx2^F/F^* mice revealed lack of IHCs without replacement by ic-OHCs. Instead, they have four to five rows of OHCs in the outer compartment and no HCs in the inner compartment (Fig. 9, A and A′).

Cochleae from *Emx2^Cre/+^; Tbx2^F/F^* mice displayed a similar reduction (but not elimination) of CD44^+^ OPCs [Fig. 9A, A′ to B′ and D; unpaired *t* test; control (14.6 ± 1.96; *n* = 3) versus *Emx2^Cre/+^; Tbx2^F/F^* (4.072 ± 1.27; *n* = 4; ***P < 0.001), in which only 28.78 ± 7.47% of cells in the OPC row express CD44] and of PROX1^+^ OPCs at P7 (Fig. 9, G and G′), and a similar replacement of many (but not all) OPCs by DC-like cells at maturity [Fig. 9, C, C′, and E; controls (13.77 ± 0.97; *n* = 4) versus *Emx2^Cre/+^; Tbx2^F/F^* (1.11 ± 1.42; *n* = 4; ****P < 0.0001; unpaired *t* test); schematically depicted in Fig. 9, H and I], as did the other embryonic *Tbx2* cKOs (Figs. 4, B, C, E, and G, 5, B to D and F) and the *Fgf8* cKOs (Fig. 3, E and E′). Hence, while IHCs induce the formation of OPCs at the expense of a default DC fate, some other signaling source must exist to account for the OPCs that still form in the absence of IHCs.

A final confirmation that IHCs induce the formation of OPCs came fortuitously in the *Emx2^Cre/+^; Tbx2^F/F^* mice. While they lack nearly all IHCs, occasionally one is detected (0.8 per cochlea, *n* = 5), presumably because, in the precursor of this cell, at least one *Tbx2^F^* allele was not ablated, and, hence, the IHC could be generated and differentiated (Fig. 9F). In these instances, a stretch of OPC-like cells was present lateral to this isolated IHC. Hence, a single IHC can induce the formation of several OPCs within a short radius of action.

**Fig. 10.**
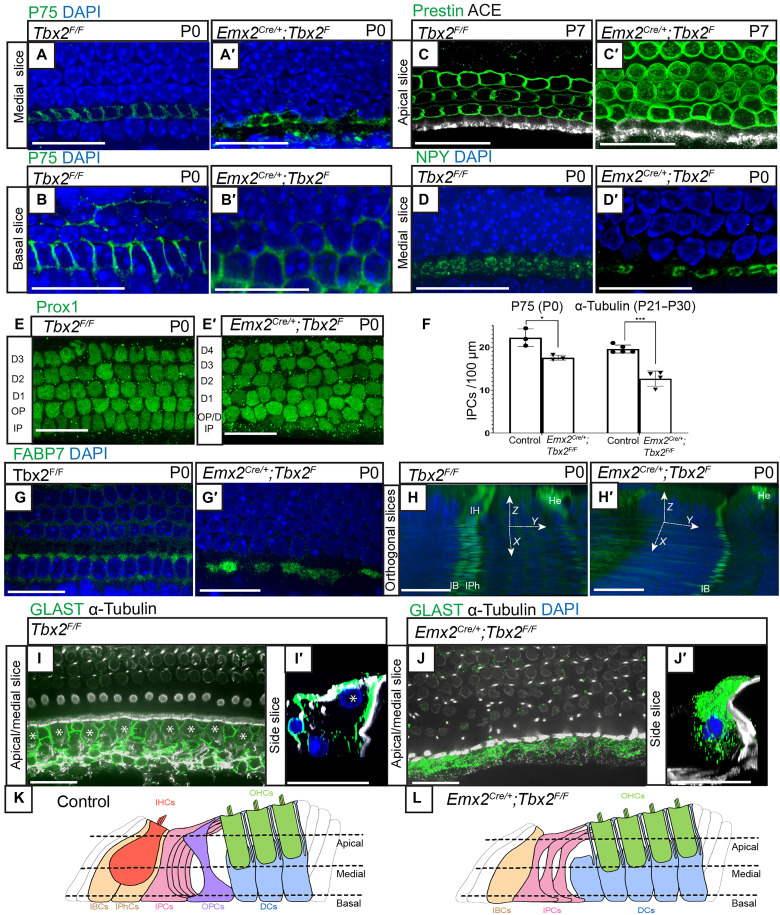
IPCs, albeit at a lower density, and IBCs form in the total absence of IHCs. (**A** to **D′**) IHCs are required for IPC high density, but not identity. P75^+^, NPY^+^, and ACE^+^ IPCs with DAPI^+^ nuclei or Prestin^+^ OHCs in neonatal cochleae of controls (*Tbx2^F/F^*) and *Emx2^Cre/+^; Tbx2^F/F^* at various depths. Controls display continuous IPCs and densely packed oblong nuclei (B). cKOs display IPC gaps at apical and middle depths but basally continuous IPCs with loosely packed cuboidal nuclei (B′). cKOs have four OHC rows (C′). (**E** and **E′**) PROX1 at P0 labels five SC rows in controls (E) and six in cKOs [four of DCs corresponding to four of OHCs + one of OPCs or DCs (OP/D) + one of IPCs (IP) with loosely packed cuboidal nuclei (E′)]. (**F**) Quantification of IPCs, labeled with P75 at P0 or α-tubulin at maturity, revealing fewer IPCs in cKOs. (**G** to **H′**) IBC and IPhC marker FABP7 on neonatal cochleae imaged through medial [(G) and (G′)] and orthogonal [(H) and (H′)] slices. Controls have two rows of FABP7^+^ IBCs and IPhCs (*yz* plane in (H)] surrounding IHCs (IH) medially and laterally [(G); and *xy* plane in (H)]. cKOs have FABP7^+^ cells in a single row despite completely lacking IHCs [(G′) and (H′)]. (**I** to **J′**) IBC and IPhC marker GLAST and α-tubulin^+^ PCs (thicker) and DCs (thinner) of mature cochlea viewed from above [(I) and (J); quantified in fig. S4)] and side [(I′) and (J′)]. [(I) and (I′)] Control, with IBCs and IPhCs wrapping IHCs (asterisks) and medial to IPCs. [(J) and (J′)] cKOs, with no IHCs but GLAST^+^ cells adjacent to IPCs. (**K** and **L**) Cell types, which, in cKOs, lack IHCs and have an extra OHCs and underlying DCs, a single row of ic-SCs (presumably, IBCs but not IPhCs), a row of apparently normal IPCs but at lower density, and most OPCs converted to DCs. Scale bars, 20 μm.

Cochleae from *Emx2^Cre/+^; Tbx2^F/F^* mice, like those of other embryonic *Tbx2* cKOs, also contained single rows of P75^+^, NPY^+^, and ACE^+^ IPCs medial to the OHCs as neonates (Fig. 10, A to D′), which formed a palisade of tubulin^+^ pillars as adults (Fig. 9, C and C′). As in other embryonic *Tbx2* cKOs, the IPCs formed a continuous line of cells at their base but were at a reduced density, as determined by P75 labeling at P0 [*Tbx2^F/F^* control (22.24 ± 2.05; *n* = 3) versus *Emx2^Cre/+^; Tbx2^F/F^* (17.57 ± 0.57; *n* = 3); *P < 0.05; unpaired Student’s *t* test; Fig. 10F] and by α-tubulin at P21 to P30 [control (19.58 ± 0.92; *n* = 5) versus *Emx2^Cre/+^; Tbx2^F/F^*(12.68 ± 1.76; *n* = 4); ***P < 0.001; Student’s *t* test; Fig. 10F]. Accordingly, the IPCs of *Emx2^Cre/+^; Tbx2^F/F^* mice were less packed nuclei and had a cuboidal, not oblong, shape (Fig. 10, B, B′, E, and E′). Hence, IHCs are required for the high density of IPCs, but IPCs still form, differentiate, and align into a row medial to the OHCs in the complete absence of IHCs.

Last, cochleae from *Emx2^Cre/+^; Tbx2^F/F^* mice, like those of other embryonic *Tbx2* cKOs, contained in their inner compartment a single row of FABP7^+^ cells as neonates (versus two rows seen in controls; Fig. 10, G to H′) and of GLAST^+^ cells as adults (Fig. 10, I to J′). These FABP7^+^ cells were reduced in numbers with respect to controls [17.67 ± 3.25 (SD) cells/100 μm in *Emx2^Cre/+^; Tbx2^F/F^* mice, *n* = 4, versus 30.00 ± 4.93 (SD) cells/100 μm in controls, *n* = 6], as was the case for other *Tbx2* cKOs [12.72 ± 0.48 (SD) cells/100 μm in *Atoh1^Cre/+^; Tbx2^F/F^* mice, *n* = 4], and, hence, we tentatively identify them as IBCs (Fig. 10, K and L). We conclude that some ic-SCs (probably IBCs and not IPhCs) are generated and aligned in a row in the inner compartment even in the complete absence of IHCs. All of the above reveal that IHCs are not required for the formation of some OPCs, IPCs, and IBCs, although they are required for the correct OPC/DC ratio, high density of IPCs, and formation of IPhCs.

## DISCUSSION

By genetically switching cell identities, converting some or all OHCs into oc-IHCs, IHCs into ic-OHCs, or not producing IHCs, we examined and tested the roles that IHCs and OHCs play in the assembly of the organ of Corti. Our results demonstrate that HC type does not determine the inner and outer identity of their surrounding SCs. Swapping the identities between IHCs and OHCs reveals that IHCs cannot induce oc-SCs to adopt the fate of an ic-SC, as SCs under oc-IHCs kept their characteristic shapes and expressed the oc-SC marker PROX1, but not the IBC/IPhC markers FABP7 or GLAST or the IPC markers P75, NPY, and ACE. HC swapping also reveals that OHCs cannot induce ic-SCs to adopt the fate of oc-SCs, as, in *Tbx2* cKOs, they expressed either IBC/IPhC or IPC markers, did not adopt the shapes of OPCs or DCs, and did not express PROX1 or the OPC marker CD44. In other words, the inner compartment and outer compartment identity of SCs in the organ of Corti is determined by signaling mechanisms that do not emanate from the respective HCs.

However, HC exchange did reveal that IHCs exert four effects on SCs (Fig. 1H): (1) formation of OPCs at the expense of DC1/2s, but not DC3s, via FGF8 signaling during a critical period of development; (2) determination of abundance, but not identity, of IPCs, also via developmentally delimited FGF8 signaling; (3) formation of presumed IPhCs, but not IBCs, during embryogenesis in an FGF8-independent manner; (4) postnatal attraction of cytoplasmic extensions from IPhC to wrap around them. These effects reveal a critical role of IHCs in orchestrating the formation and disposition of SC types with respect to IHCs and OHCs.

### Developing IHCs, via production of FGF8, control the balance of OPCs versus DCs of rows 1 and 2, but not 3

DCs of the third row (DC3s) differ anatomically from those of the first and second rows (DC1/2s) at their apical surfaces (in the reticular lamina) in that they do not intercalate between OHCs but, instead, form a barrier parallel to the third OHC row and separate them from the Hensen’s cells (HeCs) (*59*). DC3s also form a separate cluster from DC1/2s in single-cell transcriptomic examinations, revealing molecular differences between these cell subtypes (*55*). In *Insm1* cKOs, when half of the OHCs convert to oc-IHCs, most DC1/2s, but not DC3s, convert to OPCs (Fig. 2, A′, B′, C′, D, E, and F′). Even when all OHCs convert to oc-IHCs by ectopic expression of TBX2, all cells in DC1/2 positions transform into CD44^+^ OPCs, but the row of DC3 cells remains (Fig. 2, A″, B″, and F″). Clearly, IHCs can induce the conversion between OPCs and DC1/2s, but DC3s are not susceptible to this conversion.

Conversely, elimination of FGF8-producing IHCs, or simply of FGF8, produced the reverse effect: conversion of most OPCs into DCs (Figs. 3, 4, and 2, E and E′). The interconversion between OPCs and DCs was previously noted in various mutants of genes involved in FGF signaling, suggesting a model whereby FGF8 emanating from IHCs induced formation of OPCs at the expense of DCs (*33*, *39*). Our results directly test and prove this model.

As noted, FGF8 from IHCs induces differentiation of most OPCs at the expense of a DC default state. However, even in the absence of IHCs (Fig. 9) or the lack of FGF8 from IHCs and all other cochlear epithelial cells (Fig. 3, E and E′), about one-third OPCs still form (29% for *Emx2^Cre/+^; Tbx2^F/F^* and 35% for *Atoh1^Cre/+^; Tbx2^F/F^*). It follows that a signal other than epithelial FGF8, emanating from cells other than IHCs, must be in place for the induction of the remaining OPCs. Given the complete lack of OPCs upon block of FGFRs by SU5402 (Fig. 3, A and A′), this signaling mechanism must also act through FGFRs or other receptors susceptible to SU5402 blockade. Together, our results demonstrate that the developmental role of IHCs is not to determine the fate of all OPCs (because some OPCs still form in their absence) but to promote that fate and achieve a precise OPC to DC1/2 ratio, so that OPCs occupy one row and DC1/2s the next two rows.

### IHCs, via FGF8 signaling, are also responsible for the high density and compact arrangement, but not the identity, of IPCs

Another role suggested for FGF8 emanating from IHCs is the formation of IPCs (*36*, *40*, *41*). However, we find that a continuous row of IPCs forms even in the complete absence of IHCs and in the absence of FGF8 from IHCs and all other cochlear epithelial and neuronal cells (those derived from Pax2-expressing otocyst cells). IPCs appear to be properly specified because they express the three known early IPC markers (P75/NGF, NPY, and ACE) and develop the thick tubulin-filled stems characteristic of PCs. What we find instead is that, in the absence of either IHCs or FGF8, the row of IPCs loses its characteristic high density. In the cochlea, the rows of OHCs, DCs, and OPCs display equal numbers of cells, so that each DC sits under each OHC and each OPC borders its neighboring OHC in row 1. IHCs, somewhat larger, pack at a slightly lower density. On the other hand, IPCs are packed at 50% higher density than OPCs. This observation is consistent with a report showing that there were three IPCs for every two OPCs and that the intercellular distance between neighboring IPCs was ~70% of that for neighboring OPCs (*53*). In this regard, the tunnel of Corti resembles a portico with a palisade of adjacent IPCs on one side and a colonnade of evenly spaced OPCs on the other. In the absence of IHCs, IPCs form but accumulate at the same density as other cochlear rows of cells. Hence, the role of FGF8-producing IHCs is not to induce IPC identity or differentiation, but their supernumerary, dense packing. Given that FGF8 is only expressed and IHCs only generated, after all proliferation has ended in the developing organ of Corti, it follows that the role of FGF8-producing IHCs must be either to recruit more cells into the IPC row during convergence extension [which occurs between E14 and P0; (*21*)] or to promote their survival. Because of the lack of evidence for cell death in the other, less densely packed SC rows of the organ of Corti during development, the more likely interpretation is that of IHC induction of IPC recruitment. This recruitment would not come from the pools of OPCs/DC precursors, because the combined total numbers do not change in *Tbx2* cKOs (Fig. 5G), nor from IPhCs/IBCs whose total numbers are reduced, not increased, in Tbx2 cKOs (fig. S4).

Our results are not in contradiction with those of prior studies but assist their reinterpretation. The gaps in P75^+^ immunolabeling seen in *Fgf8* cKOs (Fig. 3, G′, H′, and I′) can be explained by the lower density of IPCs, which still assemble into a continuous row with wider nuclei at their base but, given the unaltered diameter of their stems, cannot form a continuous palisade at less basal positions. In addition, the complete lack of IPCs upon treatment with SU5402 (Fig. 3, C and C′) reveals that signaling susceptible to this blocker is required for their formation. However, our results reveal that this signal does not emanate from IHCs and is unlikely to be FGF8. An interesting alternative has been recently suggested with the report that β-catenin transcriptional activity, which operates as part of Wnt and other signaling pathways, is required for IPC identity (*46*).

### IHCs attract and are required in an FGF-independent manner for the formation of IPhCs, but not IBCs

The entirely unexpected results of this study are the effects of IHCs on IPhCs: generation during embryogenesis and attraction of cytoplasmic extensions. Unexpectedly, these effects do not seem to be exerted on IBCs, despite their similarity with IPhCs. We discern between IBCs and IPhCs solely on the basis of the side of IHCs that they wrap (medial by IBCs and lateral by IPhCs), as, to date, there are no molecular markers to distinguish them.

In the *Tbx2* cKOs in which IHCs convert into ic-OHCs embryonically, the only cells remaining are in the position of the IBCs, on the medial side of the HCs (Fig. 8, B, C, and F to F″). In *Emx2^Cre/+^; Tbx2^F/F^*, in which IHCs never form, we cannot use this criterion, but there is a reduction of the total number of labeled cells, and the simplest interpretation is that IPhCs are missing, while IBCs remain. Either IPhCs are not generated in the absence of IHCs or they disappear during embryonic development (die or dedifferentiate and move away) before we can detect them. It has been suggested on the basis of the similarity of their molecular profiles that some IBCs/IPhCs are recruited from the medially adjacent cells of the Kölliker’s organ (*4*). Furthermore, in the neonatal cochlea, ablated IBCs/IPhCs are replaced by Kölliker’s organ cells that move toward the IHCs and transdifferentiate into IBCs/IPhCs, a process that requires the presence of IHCs (*60*). It is tempting to speculate that signaling from IHCs recruits Kölliker’s organ cells to become IPhCs and that in the absence of IHCs (or when converted to ic-OHCs), this recruitment fails. Whatever this signaling is, it is not FGF8, as both IBCs and IPhCs form in its absence (Fig. 3, F and F′).

Last, as interesting as the SCs that are induced by IHCs, are those that are independent of HCs in the cochlea. Besides IBCs, DC3s (Fig. 2), HeCs (also labeled with anti-FABP7; fig. S5), and Claudius cells (CCs; also labeled with CD44; Figs. 1, B to B″; 2, E and E′; and 3, A to D) were normal in appearance and density in all the mutants examined. It appears that IHCs exert effects on the development of IPhCs, IPCs, OPCs, and DC1 and DC2, but not on cells delimiting the organ of Corti: IBCs on its medial edge and DC3s, HeCs, and CCs on its lateral edge.

### Targeted cell conversions as a method for elucidating the role of individual cell types in organ development

Here, we implemented a methodology for the study of cell types in the formation of organs that consists in converting cells of one type into another at chosen developmental stages to elucidate what role that cell type plays in the formation of the organ. The approach is conceptually analogous to the classical transplantations that led to the discovery of multicellular embryonic organizers (*44*, *45*). However, the genetic methodology that we use, while also intended to reveal non–cell-autonomous effects, offers the advantage of targeting cells of a single type that are embedded amidst other cells within an organ, something that surgical transplantations cannot accomplish.

In our case, the approach is made possible by the discovery that IHCs and OHCs can switch identities (*26*–*28*), something that we have learned can happen between other cell types (e.g., OPCs and DCs). Hence, while this study is limited to cochlear IHCs and OHCs, other cell types will follow in the organ of Corti as well as in other organs. Our study is also greatly facilitated by the precise distribution of cells in the organ of Corti, in which even alterations of a single cell can be detected. However, although more challenging, studies of this sort should be pursued in other organs. What will be needed is the identification of genetic manipulations that, like those of *Tbx2* and *Insm1* in cochlear HCs, trigger the conversion between other cell types.

### Additional considerations

It may be argued that, because TBX2 is expressed by both inner HCs and SCs (including IBCs and IPhCs), the effects of the *Emx2^Cre^* ablation, which targets both, may directly affect the development of ic-SCs in a cell-autonomous manner. It has been suggested that removal of TBX2 from ic-SCs results in their developing as oc-SCs (i.e., PROX1^+^ cells) (*29*). We, however, do not observe this effect. In *Emx2^Cre/+^; Tbx2^F/F^* neonates PROX1, a marker of oc-SCs, labels the nuclei of DCs (four rows because of the extra row of OHCs) and PCs, but not the nuclei of cells in the inner compartment medial to the IPCs (Fig. 10, E and E′). Furthermore, in *Emx2^Cre/+^; Tbx2^F/F^* mice, IPCs form and cells medial to them still express the IBC/IPhC markers FABP7 and GLAST (Fig. 10, G′, J, and J′). The reduction of the number of these cells happens as well when TBX2 is removed from HCs and not SCs (in the *Atoh1^Cre^*, *Gfi1^Cre^*, and *Fgf8^CreER^* cKOs) and is, therefore, accounted for by the lack of IHCs, not because of a cell-autonomous requirement of TBX2 in ic-SC fate.

The phenotype of *Emx2^Cre/+^; Tbx2^F/F^* mice, with no IHCs generated at all and an extra row or two of OHCs, bear major consequences regarding how TBX2 regulates HC development. Removal of TBX2 from nascent IHCs, as we have seen with *Atoh1^Cre^*, *Gfi1^Cre^*, and *Fgf8^CreER^* drivers, makes them develop as OHCs, while ectopic expression in OHCs does the reverse (*28*–*30*). Hence, the presence or absence of TBX2 determines whether a cochlear HC will become an IHC or an OHC. The fact that IHCs do not form when TBX2 is absent before their precursors exit the cell cycle reveals an earlier requirement for TBX2 in the generation, not just the fate, of IHCs. How and why in *Emx2^Cre/+^; Tbx2^F/F^* mice IHCs are not formed but display an extra row of OHCs are subjects of ongoing investigations but beyond the scope of this study, which focuses on the developmental effects of IHCs and OHCs on SCs. For the present work, the *Emx2^Cre/+^; Tbx2^F/F^* mice provide a model for determining the effects on SCs of lacking IHCs.

## MATERIALS AND METHODS

### Mouse lines

All animal care and procedures were in strict accordance with the *Guide for the Care and Use of Laboratory Animals* published by the National Institutes of Health (NIH) and were approved by Northwestern University’s Institutional Animal Care and Use Committee (animal study protocols IS00021813 and IS00017716). Mice were group housed with food and water provided ad libitum under a 12-hour light/12-hour dark cycle and temperatures of 18° to 23°C with 40 to 60% humidity.

The floxed *Insm1^F^* mouse allele (*48*) was bred in the C57BL/6J background. The floxed *Tbx2^F^* mouse allele (*61*), provided by V. Christoffels (Amsterdam University Medical Center), was bred in the FVB background. The floxed *Fgf8^F^* mouse allele (*62*), provided by M. Lewandoski (NIH), was bred in an undetermined background. Regarding the knock-in mouse lines used for this study, the *Atoh1*-Cre knock-in mouse line (*63*) was bred from a mixed background of CD1 and C57BL/6. The *Gfi1*-Cre knock-in mouse line (*64*) was bred from a C57BL/6 background. The *Fgf8*-CreER knock-in mouse line (*65*), provided by A. Moon and M. Deans (University of Utah), was bred from a mixed 129Sv and C57BL/6J background. The Tg*Pax2*-Cre transgenic mouse line (*54*), provided by A. Groves (Baylor College of Medicine), was bred in a mixed CD1xFVBxFVBsi background. The *Emx2*-Cre knock-in mouse line, provided by RIKEN Bioresource Center (acc. no. CDB0020K), was bred in the C57BL/6 background and the R26-TAT mouse line (*30*) provided by the Jackson Laboratory, was bred in the mixed background C57BL/6 x DBA/2J. The knock-in mouse lines *Atoh1*-cre, *Gfi1*-cre, and *Fgf8*-CreER breed with the floxed *Tbx2^F^* mouse allele were backcrossed several generations into an FVB background.

For the conditional ablation of *Tbx2*, we injected *Fgf8*^CreER^; *Tbx2*^F/F^; R26^LSL-tdTomato/+^ and *Tbx2*^F/F^; R26^LSL-tdTomato/+^ littermate controls intraperitoneally at E14.5 + E15.5, P0, P3, or P7 with a single dose of tamoxifen (80 mg/kg; Sigma-Aldrich, T5648) freshly solubilized in corn oil (20 mg/ml stock) using a Hamilton syringe and a 27-gauge needle. All mice used in this study were generated by intercrossing the above mouse lines. Similar numbers of male and female animals were used for all analyses at the ages indicated in the figure legends.

### Whole-mount cochlear preparations

Inner ears were collected at P0, P4, and P7 and fixed in 4% paraformaldehyde (PFA) for 2 hours at 4°C or at P21 and older where the animals were perfused with 4% PFA and the inner ears were collected and placed in 4% PFA overnight at 4°C. After fixation, the tissues obtained after P7 were decalcified with 10% EDTA (pH 7.4) from 1 to 72 hours depending on age until the bone was soft enough for cochlear microdissection. To expose sensory epithelia for whole-mount immunofluorescence, early postnatal cochleae (from P0 to P7) were microdissected in one piece and mature cochleae (P21 and older) into four segments (one apical, two medial, and one basal).

### Immunofluorescence

Cochleae were immunostained using two methods, freeze-thaw or antigen retrieval, and with or without postfixation in 4% PFA for 30 min before starting the staining, as indicated in the “key resources table” (table S1). The freeze-thaw method consisted of permeabilizing the tissues, previously incubated with 30% sucrose for 20 min, by freezing at −80°C for 7 min followed by thawing at room temperature for 10 min. The antigen retrieval method consisted of incubating the tissues in 10 mM sodium citrate and 0.25% Triton X-100 (pH 6) for 20 min at 92°C and then cooling to room temperature. In both methods, the tissues were then washed three times with 1× phosphate-buffered saline (PBS) for 5 min followed by incubation with blocking solution (10% normal donkey serum in tris-buffered saline with 1% Triton X-100) for 1 hour at room temperature. After incubation, the primary antibodies found in the key resources table (table S1) diluted in blocking solution were added to the cochlea for overnight incubation at 4°C. On the next day, the tissues were washed three times in 1× PBS and incubated with secondary antibodies diluted in blocking solution as indicated in the key resources table (table S1) for 1 to 2 hours at room temperature. For the last step, tissues were washed with 1× PBS, and nuclei were counterstained with 4′,6-diamidino-2-phenylindole (20 μg/ml) in 1× PBS for 15 min before a final wash and mounting in ProLong Gold antifade mounting medium (Thermo Fisher Scientific).

### In situ hybridization

Embryos at E15.5 and E17.5 were collected from timed pregnant females (vaginal plug = E0.5), and their cochleae were extracted and fixed with 4% PFA for 24 hours followed by incubations in sucrose gradients (5, 10, and 20%) for cryoprotection and then mounted in optimal cutting temperature compound (Thermo Fisher Scientific) and frozen on dry ice. The cochleae were serially sectioned at 14-μm thickness, mounted on SuperFrost Plus slides (Thermo Fisher Scientific), and dried briefly at room temperature before storage at −80°C. The manufacturer’s standard protocol for chromogenic in situ hybridization (RNAscope 2.5 HD Red Assay, Advanced Cell Diagnostics) was followed using the *Fgf8* probe (313411) (Advanced Cell Diagnostics) (table S1). In situ hybridization was followed by immunofluorescence as described before, without freeze-thaw or antigen retrieval steps, with anti-myosin VIIa and Alexa Fluor 488 donkey anti-rabbit antibodies [see the key resources table (table S1)].

### Cochlear explant culture and inhibition of FGF

Cochlear explants were isolated from *Atoh1^Cre/+^; Insm1^F/F^* and control *Insm1^F/F^* littermates at E17.5. For each sample, the cochlear roof and any excess connective tissue were removed to expose the sensory epithelium (*66*), which was then transferred to a plastic culture dish containing fresh culture medium [Dulbecco’s modified eagle’s medium/F12 (1:1) supplemented with 10% fetal bovine serum and ampicillin (50 μg/ml)]. The explants were cultured for 6 days at 37°C with 5% CO_2_. FGFR signaling was inhibited by SU5402 (Calbiochem, San Diego, CA) (table S1) (*49*). A stock solution of SU5402 was dissolved in dimethyl sulfoxide (DMSO) and then diluted to a concentration of 10 μM in culture medium or an equivalent amount of DMSO as control, added on day 1 (E18.5), day 2 (E19.5), and day 3 (E20.5) of culture. Following the culture period of 6 days, explants were fixed in 4% PFA for 30 min at room temperature and then processed for immunofluorescence using the antigen retrieval method described above.

### Image acquisition and analysis

Images of cochlear whole mounts and organotypic cultures were acquired as before (*67*) using a Yokogawa CSU-W1 spinning disk confocal microscope on a Nikon Ti2 microscope with a Hamamatsu Flash 4.0 V3 camera operated by NIS-Elements. An Apo TIRF λ100× oil differential interference contrast objective was used with a numerical aperture of 1.49 using a step size of 0.2 μm, image size of 2048 × 2044, and pixel size of 0.06 μm per pixel. For imaging cultures, a Plan Apo λ 60× oil objective with a numerical aperture of 1.4 was used with a step size of 0.3 μm, image size of 1024 × 1024, and pixel size of 0.09 μm per pixel. Exposure times were set to ensure high signal-to-noise ratio and no saturation in the image. Gain and offset adjusting were performed to ensure that no saturated or undersaturated pixels were present. Identical capture and analysis conditions were used for each experimental and control tissue. Images were processed and three-dimensional renderings were generated using NIS-Elements.

### Quantification and statistical analysis

Statistical analysis was performed using GraphPad Prism 10.2.0 software (GraphPad software Inc., San Diego, CA, USA). Normality was assessed using Shapiro-Wilk and Kolmogorov-Smirnov tests. If data passed this test, then an unpaired *t* test or ANOVA was performed where appropriate (two datasets versus multiple datasets, respectively). Relevant information for each experiment including sample size, statistical tests, and *P* values is described in the figure legends. In all cases, *P* < 0.05 is considered statistically significant.

Quantification of OPCs was performed at P7 on CD44-stained samples by counting the number of CD44^+^ cells in the OPC row per 100 μm and between P21 and P51 on α-tubulin–stained samples by counting the number of OPC and DC identified by their unique morphology in the OPC row per 100 μm or in the three rows of DCs plus the row of OPCs.

Quantification of IPCs was performed at P0 on P75-stained samples by counting the number of P75^+^ cells in the IPC row per 100 μm (measured at the base of the cell and along the cochlear spiral) and between P21 and P51 on α-tubulin–stained samples by counting the number of IPCs identified by their unique morphology in the IPC row per 100 μm. Quantification of IBCs/IPhCs was performed at P21 to P51 on GLAST-stained samples by counting the number of nuclei of GLAST^+^ cells surrounding the IHCs or ic-OHCs per 100 μm. Unless otherwise noted, quantifications were performed in samples taken from the mid-basal turn of the cochlea of at least three different animals. Each sample was obtained from a different animal, and, therefore, all samples are biological replicates.

### Hearing tests

DPOAEs were measured at 3 to 8 weeks of age in *Tbx2^F/F^*, *Atoh1^Cre/+^; Tbx2^F/F^*, *Gfi^Cre/+^; Tbx2^F/F^*, and *Fgf8^CreER/+^; Tbx2^F/F^* injected with tamoxifen at E14.5/15.5 or P1 as previously described (*26*, *48*, *68*). DPOAEs were acquired using an ER-10B^+^ low-noise DPOAE microphone (Etymotic Research) coupled to a custom emission probe designed and fabricated by J. H. Siegel (Northwestern University) that fit securely in the ear canal. Calibrations were determined for each individual mouse by measuring the stimulus pressures in the ear canal using the emission microphone. DPOAEs were generated using a two-tone input with a frequency ratio of *f*2/*f*1 = 1.2. Responses were analyzed using Emission Averager (*69*). These results are provided as iso-input functions, in which the parameter is stimulus frequency, and when the levels of primaries, *f*1 and *f*2, were both set at 70-dB sound pressure level. Means ± SEMs are plotted for each group. Statistical significance was determined in Microsoft Excel using the Student’s *t* test: two-tailed distribution and two samples assuming unequal variance. A *P* value of less than 0.05 was determined to be statistically significant. Each DPOAE was obtained from a different animal, and, therefore, all measurements are biological replicates.
