# Supplementary material for: Targeted cell interconversions reveal inner hair cell control of organ of Corti cytoarchitecture
Source: Sci Adv. 2025 Oct 29;11(44):eadz3944. doi: 10.1126/sciadv.adz3944 (PMC12571072; doi:10.1126/sciadv.adz3944)
Supplement: Supplementary file 1 — Figs. S1 to S5 Table S1 Legends for movies S1 and S2 [file sciadv.adz3944_sm.pdf]

Science Advances
AAAS

Supplementary Materials for

Targeted cell interconversions reveal inner hair cell control of organ of Corti cytoarchitecture

Ignacio García-Gómez *et al.*

Corresponding author: Jaime García-Añoveros, anoveros@northwestern.edu

Sci. Adv. **11**, eadz3944 (2025)
DOI: 10.1126/sciadv.adz3944

The PDF file includes:

Figs. S1 to S5
Table S1
Legends for movies S1 and S2

Other Supplementary Material for this manuscript includes the following:

Movies S1 and S2

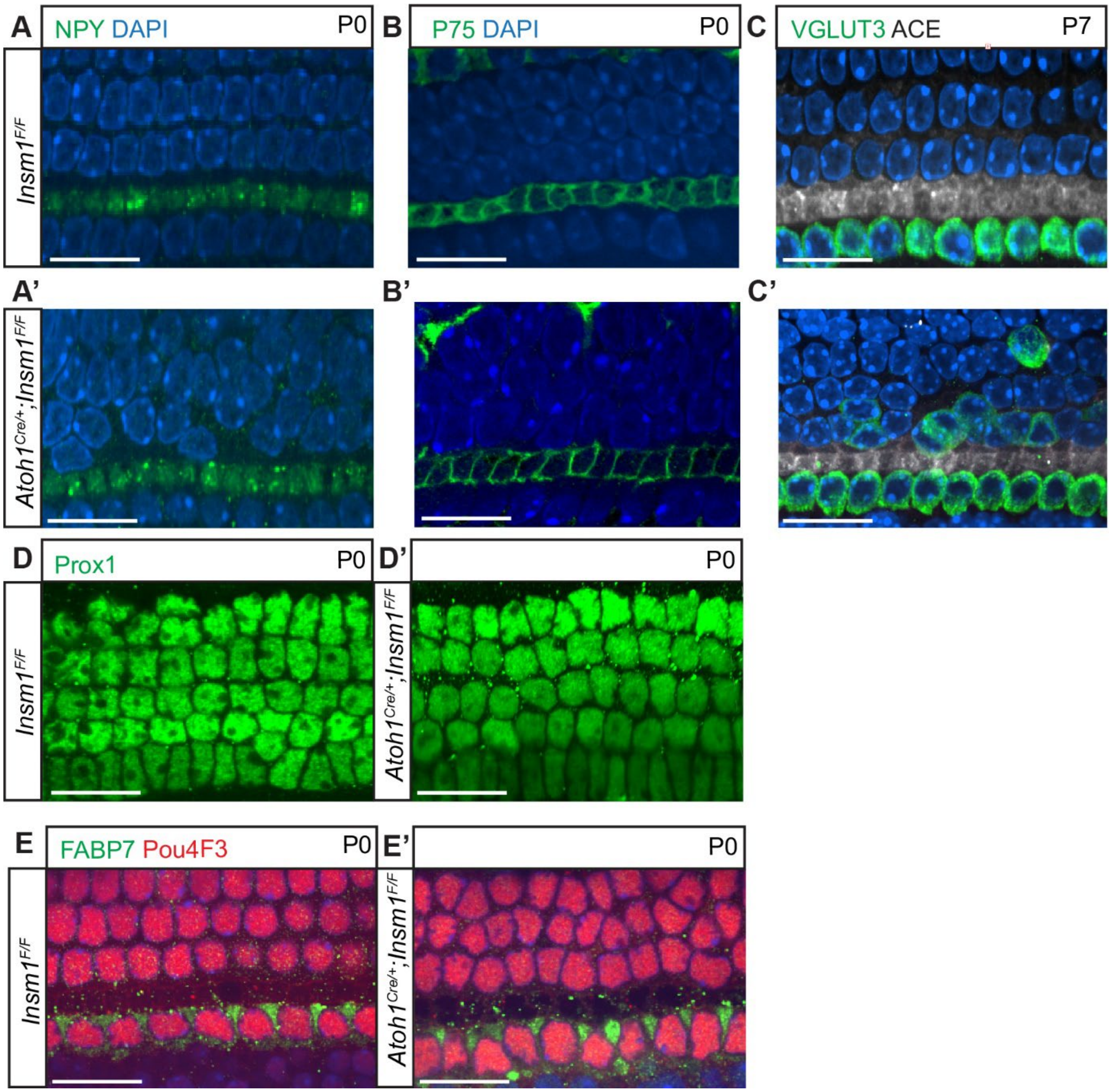

Fig. S1. Inner and outer compartment supporting cells in neonatal Insm1 cKOs (*Atoh1$^{Cre/+}$; Insm1$^{F/F}$*). (**A** to **C'**) Immunolabelings of NPY [green; (A,A')], P75/NGFR [green; (B,B')] and Angiotensin I Converting Enzyme (ACE) [white; (C,C')] reveal a single row of IPCs in cKOs [*Atoh1$^{Cre/+}$; Insm1$^{F/F}$* (A',B',C')], as in controls [*Insm1$^{F/F}$* (A,B,C)]. DAPI (blue) labels nuclei and VGLUT3 (green) labels IHCs and oc-IHCs. (**D,D'**) Immunolabeling of PROX1 (green) reveals the normal 5 rows of IPC, OPC and DC1-3 supporting cell nuclei in cKOs [*Atoh1$^{Cre/+}$; Insm1$^{F/F}$* (D')] also seen in controls [*Insm1$^{F/F}$* (D)]. In both cases, the row of IPCs (bottom) has a higher density of nuclei, with their characteristic oblong shape. By comparison, the nuclei of OPCs and DCs are less densely packed and cuboidal. (**E,E'**) Immunolabeling of FABP7 (green) reveals IBCs and IPhCs around IHCs in both controls [*Insm1$^{F/F}$* (E)] and cKOs [*Atoh1$^{Cre/+}$; Insm1$^{F/F}$* (E')], but not in the outer compartment. POU4F3 (red) labels hair cell nuclei. All scale bars are 20 μm.

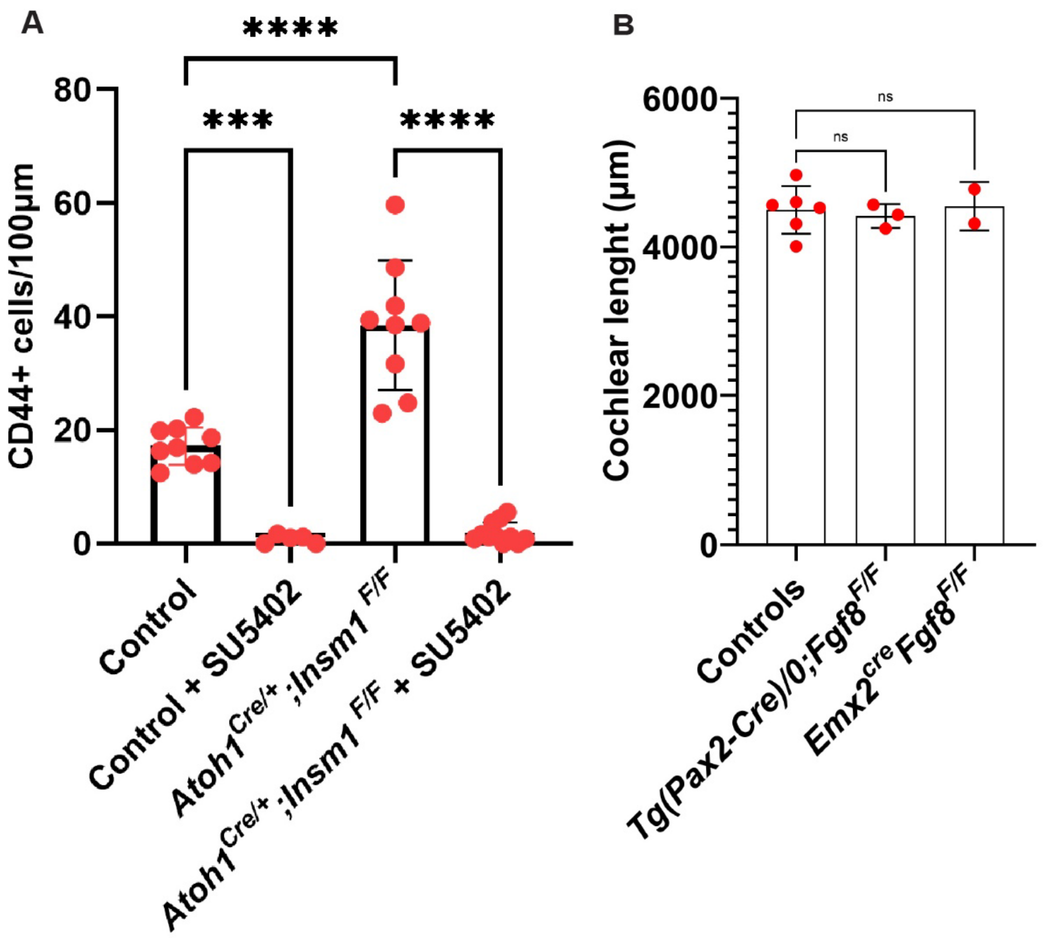

Fig. S2. Quantification supplementary to Figure 2. (A) Quantification of CD44+ cells in organotypic cochlear explants reveals a significant increment of OPCs in *Atoh1*$^{Cre/+}$*; Insm1*$^{F/F}$ (n=9; 38.51 ± 11.39 cells/100µm) *vs Insm1*$^{F/F}$ (control) littermates (n=9; 17.23 ± 3.3 cells/100µm); ****p<0.0001. However, exposure to FGFR inhibitor SU5402 eliminated CD44+ OPCs in both control and *Atoh1*$^{Cre/+}$*; Insm1*$^{F/F}$: Control (n=9; 17.23 ± 3.3 cells/100µm) vs Control + SU5402 (n=5; 0.79 ± 0.7663 cells/100µm); ***p<0.001 and *Atoh1*$^{Cre/+}$*; Insm1*$^{F/F}$ (n=9; 38.51 ± 11.39 cells/100µm) *vs Atoh1*$^{Cre/+}$*; Insm1*$^{F/F}$ + SU5402 (n=11; 1.91 ± 1.86 cells/100µm); ****p<0.0001. The number of CD44+ OPCs was analyzed by one-way ANOVA with Tukey's multiple comparison test.

(B) Quantification of cochlear length at P0 reveals no difference in length of *Fgf8* cKOs compared to controls: *Tg(Pax2-Cre)/0; Fgf8*$^{F/F}$ (n=3) *vs Fgf8*$^{F/F}$ controls (n=6) (4416 ± 160.2 µm *vs* 4496 ± 320.3 µm); *ns* P>0.05 and *Emx2*$^{Cre/+}$*; Fgf8*$^{F/F}$ (n=2) *vs Fgf8*$^{F/F}$ controls (n=6) (4547 ± 325.4 µm *vs* 4496 ± 320.3 µm); *ns* P>0.05. Cochlear length was analyzed by one-way ANOVA with Dunnett correction for multiple comparisons.

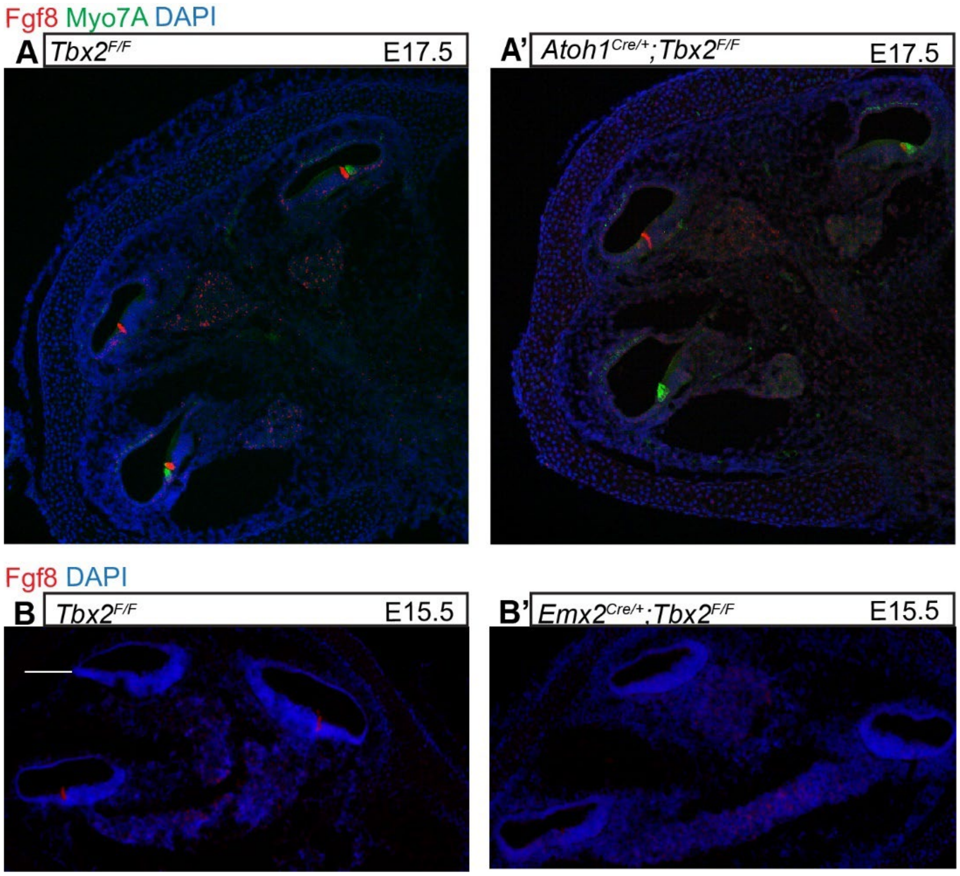

Fig. S3. ***Fgf8*** **is transiently expressed in nascent IHCs of** ***Atoh1**$^{Cre/+}$**; Tbx2**$^{F/F}$* **embryos, but not in** ***Emx2**$^{Cre/+}$**; Tbx2**$^{F/F}$* **embryos.** (**A**, **A'**) *In situ* hybridization for *Fgf8* mRNA (red) plus immunolabeling for hair cell marker MYO7A (green), with a nuclear DAPI counterstain (blue) on sections of E17.5 cochlea from: (A) *Tbx2*$^{F/F}$ (control), in which IHCs of all cochlear turns express *Fgf8* [N=32/32 IHCs that express *Fgf8*: 8 in apical, 8 in mid apical, 8 in mid basal, and 8 in basal turns of the cochlea]; (A') *Atoh1*$^{Cre/+}$*; Tbx2*$^{F/F}$, in which some IHCs express FGF8 in apical, but none in basal, cochlear turns [N=9/24 IHCs that express *Fgf8*: 5/8 in apical, 4/8 in middle, and 0/8 in basal turns of the cochlea]. *Fgf8* expression levels are lower in middle than apical turns. (**B**,**B'**) *In situ* hybridization for *Fgf8* mRNA (red) with a nuclear DAPI counterstain (blue) on sections of E15.5 cochleae from: (B) *Tbx2*$^{F/F}$ (control), with FGF8-expressing IHCs in middle and basal turns of the cochlea, but no FGF8-expressing cells in the apical turn, in which hair cells have not yet been produced [N=37/57 sections with FGF8-expressing cells: 0/19 in apical, 18/19 in middle, and 19/19 in basal turns of the cochleae from 3 embryos]; (B') *Emx2*$^{Cre/+}$*; Tbx2*$^{F/F}$, with no cells in 48 sections across the organ of Corti expressing *Fgf8* [apical (0/15), middle (0/17) and basal (0/16) turns of the cochlea from 3 embryos were examined]. Scale bar (100 μM) applies to all panels.

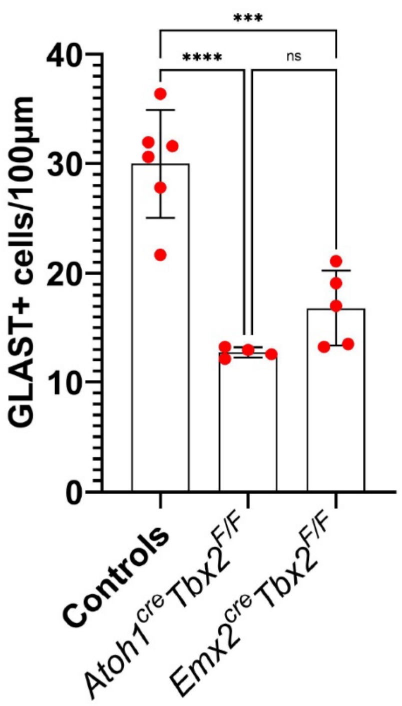

Fig. S4. Quantification supplementary to Figure 7 and Figure 9. Quantification of GLAST+ cells (IBCs/IPhCs) reveals a reduction of IBCs/IPhCs, expressed as number of cells per 100µm, in *Atoh1*$^{Cre/+}$*; Tbx2*$^{F/F}$ (n=4) *vs* controls (n=6) (12.72 ± 0.48 *vs* 30.00 ± 4.93; ****p<0.0001) and in *Emx2*$^{Cre/+}$*; Tbx2*$^{F/F}$ (n=5) *vs* controls (16.79 ± 3.44 *vs* 30.00 ± 4.93; ***p<0.001). The number of GLAST+ cells was analyzed by one-way ANOVA with Dunnett correction for multiple comparisons.

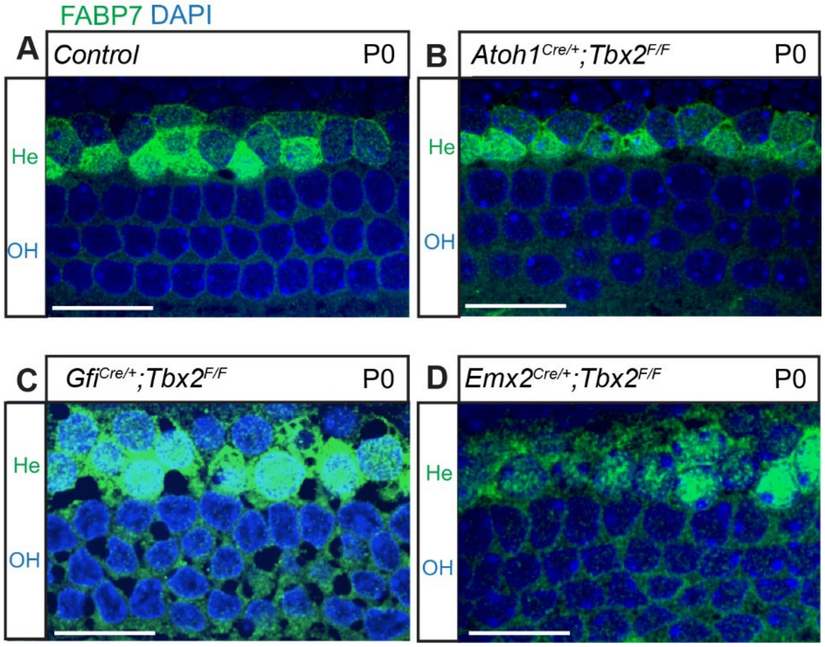

Fig. S5. Lack of IHCs or their conversion to ic-OHCs does not affect development of Hensen Cells. (**A** to **D**) Immunolabeling of FABP7 (green), a marker of Hensen Cells (He) examined at P0, in the organs of Corti. Controls (A) showed clearly the Hensen Cells (FABP7+ cells) on top of the rows of OHCs. A similar pattern of staining was observed in all the *Tbx2$^{F/F}$* cKO: *Atoh1$^{Cre/+}$; Tbx2$^{F/F}$* (B), *Gfi$^{Cre/+}$; Tbx2$^{F/F}$* (C), and *Emx2$^{Cre/+}$; Tbx2$^{F/F}$* (D).

Table S1. Key Resources Table

REAGENT	SOURCE	IDENTIFIER
**Primary antibodies (dilution)**		
**Goat anti-ACE/CD143 (1:50)**	Biotechne	Cat #AF1513
**Goat anti-Oncomodulin (1:200)**	Santa Cruz	Cat #SC-7446
**Goat anti-Parvalbumin (1:2000)**	Swant	Cat #PVG-213
**Goat anti-SOX2 (1:500)**	Santa Cruz	Cat #SC-17320
**Mouse anti-Brn-3c (POU4F3) (1:100)**	Santa Cruz	Cat #SC-81980
**Mouse anti-Calb2 (1:100)**	Proteintech	Cat #66496-1-Ig
**Mouse anti-α-tubulin (1:400)**	Sigma-Aldrich	Cat #T6199
**Rabbit anti-BLBP (FABP7) (1:200)**	Abcam	Cat #AB32423
**Rabbit anti-EAAT1 (GLAST) (1:100)**	Abcam	Cat #AB416
**Rabbit anti-MyosinVIIa (1:400)**	Proteus Biosciences	Cat #25-6790
**Rabbit anti-NPY (1:400)**	Biocompare	Cat #T-4069
**Rabbit anti-p75 (1:100)**	Millipore	Cat #AB1554
**Rabbit anti-Prestin (1:1000)**	Northwestern University (J Zheng)	-
**Rabbit anti-PROX1 (1:500)**	Millipore	Cat #AB5475
**Rabbit anti-Vglut3 (1:500)**	Synaptic Systems	Cat #135-203
**Rat anti-CD44 (1:100)**	BD Pharmingen	Cat #550538
**Secondary antibodies (dilution)**		
**Alexa Fluor 488 Donkey anti-rabbit (1:100)**	Jackson InmunoResearch	711-545-152
**Alexa Fluor 488 Donkey anti-rat (1:100)**	Jackson InmunoResearch	711-585-153
**Alexa Fluor 594 Donkey anti-goat (1:100)**	Jackson InmunoResearch	705-586-147
**Alexa Fluor 594 Donkey anti-mouse (1:100)**	Jackson InmunoResearch	711-585-150
**Alexa Fluor 594 Donkey anti-rat (1:100)**	Jackson InmunoResearch	712-586-153
**Alexa Fluor 647 Donkey anti-goat (1:100)**	Jackson InmunoResearch	705-606-147
**Alexa Fluor 647 Donkey anti-mouse (1:100)**	Jackson InmunoResearch	711-605-151
**Alexa Fluor 568 Donkey anti-goat (1:100)**	Invitrogen	Cat #A-11057
**Other**		
***Fgf8*-probe**	Advanced Cell Diagnostics RNAscope	Cat #313411
**SU5402**	Abcam	Cat #AB146602

Movie S1: 3D visualization of the confocal projection (same as in figure 8E) of *Tbx2*$^{F/F}$ (control) reveal GLAST+ cytoplasmic extensions (green) wrapping around medial and lateral sides of Calb2+ IHCs (red).

Movie S2: 3D visualization of the confocal projection (same as in figure 8F) of *Atoh1*$^{Cre/+}$*;* *Tbx2*$^{F/F}$ reveal GLAST+ cytoplasmic extensions (green) wrapping around medial but not the lateral sides of OCM+ ic-OHCs (orange).
